# Proteome-Level Autophagy–Lysosome Remodelling Marks Ageing in Human Dermal Fibroblasts and Nominates Hydroxytyrosol as a Candidate Nutraceutical

**DOI:** 10.3390/ijms27156808

**Published:** 2026-07-29

**Authors:** Meng Cai, Meihong Xu

**Affiliations:** 1 Department of Nutrition and Food Hygiene, School of Public Health, Peking University, Beijing 100191, China; 1810306215@pku.edu.cn; 2 Beijing Key Laboratory of Toxicological Research and Risk Assessment for Food Safety, Peking University, Beijing 100191, China

**Keywords:** skin ageing, dermal fibroblast, proteostasis, autophagy–lysosome, network medicine, hydroxytyrosol

## Abstract

Autophagy–lysosome dysfunction accompanies dermal fibroblast ageing, yet whether remodelling is transcriptional or post-transcriptional in primary human cells remains unresolved. We reanalysed the Genetic and Epigenetic Signatures of Translational Ageing Laboratory Testing(GESTALT) paired RNA sequencing (RNA-seq) and tandem mass tag (TMT) proteome from 82 donors (aged 22–89) using Data Integration Analysis for Biomarker discovery using Latent cOmponents (DIABLO) for supervised multi-omics integration, weighted gene co-expression network analysis (WGCNA), external Genotype-Tissue Expression(GTEx) transcriptomic comparison, network medicine proximity mapping and CDOCKER molecular docking. Three analyses converged on the autophagy–lysosome axis: Kyoto Encyclopaedia of Genes and Genomes (KEGG) Lysosome ranked first in discordant-quadrant analysis; gene set enrichment analysis (GSEA) identified vacuole organisation and macroautophagy as the top age-upregulated Gene Ontology (GO) terms; and WGCNA recovered KEGG Lysosome in the brown module. Module regression localised most proteomic age signals to the lysosomal degradative-capacity module, whereas the proteasome was unaffected. McNemar testing and GTEx comparison supported a protein-side, post-transcriptional origin. TCIRG1, CTSA and ATP6V0D1 were recurrent hubs. Network proximity computationally prioritised hydroxytyrosol as a lysosomal-degradative-capacity-preferential candidate, and CDOCKER on cathepsin A linked its advantage over tyrosol to an ortho-hydroxyl group forming additional hydrogen bonds. These results support protein-layer-dominant autophagy–lysosome remodelling as a feature of dermal fibroblast ageing and suggest a cell-type-resolved computational route from ageing proteomics to testable dietary candidates.

## 1. Introduction

Dermal fibroblasts maintain skin integrity through continuous synthesis and remodelling of the collagen-rich extracellular matrix. Their dysfunction drives dermal thinning, ECM dysregulation and chronic inflammation in aged skin [1]. Most molecular research on fibroblast ageing has centred on the extracellular consequences: collagen fragmentation, matrix metalloproteinase overexpression and the senescence-associated secretory phenotype. Their intracellular proteostasis programmes have received less attention. Recent proteomic surveys have begun to map age-associated proteome remodelling in primary dermal fibroblasts [2,3], with one of these cohorts also profiled by RNA sequencing on the same donor preparations [4], enabling direct mRNA-protein comparison. The relative contribution of transcriptional versus post-transcriptional control to this remodelling has not been resolved at the module level.

The 2023 update of the hallmarks of ageing elevated disabled macroautophagy from a sub-category of proteostasis to a standalone hallmark alongside the long-recognised loss of proteostasis [5]. Both hallmarks converge on the autophagy–lysosome system. Lysosomal biogenesis and degradative throughput are coordinated by the transcription factor EB (TFEB)-driven Coordinated Lysosomal Expression and Regulation (CLEAR) transcriptional programme [6], whose nuclear activity is gated by mTORC1-mediated phosphorylation under nutrient-replete conditions [7]. In senescent human fibroblasts, Carroll et al. identified that mTORC1 is constitutively active and resistant to both serum and amino acid starvation, with persistence linked to primary cilia defects and elevated intracellular amino acid levels [8]. Smith and Carroll have since highlighted the underappreciated role of mTORC1 signalling and the autophagy–lysosome axis in skin senescence [9]. The evidence base, however, rests primarily on experimentally induced senescence. Whether this axis undergoes measurable remodelling during chronological ageing of primary cells remains untested at the systems level.

Paired transcriptome–proteome studies have shown that mRNA and protein abundances diverge progressively with age. Ori et al. attributed age-related proteome changes in rat brain and liver primarily to altered translation output rather than transcript abundance alone [10]. In primate prefrontal cortex, Wei et al. documented substantial mRNA–protein decoupling specific to ageing rather than development [11]. More recently, Di Fraia et al. traced age-dependent proteome remodelling in the killifish brain to aberrant translation elongation, with widespread depletion of basic amino acid-enriched proteins occurring independently of transcript changes [12]. Ding et al. then mapped this erosion across 13 human tissues and 516 samples from donors aged 14 to 68 years [13]. Two recent reviews have synthesised these observations under the framework of age-dependent translational constraints [14,15]. All of these studies analysed bulk tissue. Cell-type composition shifts with age are a well-recognised confounder at that resolution. The GESTALT cohort (Genetic and Epigenetic Signatures of Translational Aging Laboratory Testing) addresses this gap at the dermal fibroblast level. Primary dermal fibroblasts from 82 healthy donors aged 22–89 years have been profiled by paired RNA sequencing and tandem mass tag proteomics from the same cell preparations [3,4].

Network medicine maps disease-associated proteins to connected modules in the human interactome [16], with closest-distance proximity to compound target sets predictive of therapeutic effects across diverse disease areas [17]. The framework has been applied to polyphenols, with network proximity predictive of their known therapeutic effects [18]. Existing ageing applications use pan-tissue gene sets. These analyses lack cell-type resolution, and structural docking has rarely been combined with network proximity for dietary-compound prioritisation. Whether cell-type-specific ageing modules can serve as a basis for prioritising compounds already present in the human diet remains an open question.

We reanalysed the GESTALT paired RNA-seq and TMT proteomic profiles to ask whether the autophagy–lysosome axis is selectively remodelled at the protein layer during chronological ageing of primary dermal fibroblasts, whether complementary analytical strategies converge on the same molecular hubs, and whether the resulting target module can guide nutraceutical prioritisation under structural docking validation. The analysis combined supervised multi-omics integration, predefined-module-targeted regression, unsupervised protein co-expression, external GTEx transcriptomic comparison, network medicine compound mapping and CDOCKER molecular docking. The data support protein-layer-dominant autophagy–lysosome remodelling as a tractable feature of skin fibroblast ageing, with hydroxytyrosol nominated as a lysosomal-degradative-capacity-preferential lead and a single-hydroxyl structure–activity contrast linking network topology to protein–ligand docking energetics.

## 2. Results

### 2.1. Pervasive mRNA–Protein Decoupling with Autophagy-Selective Coupling Decline in Ageing Fibroblasts

**Figure 1:**
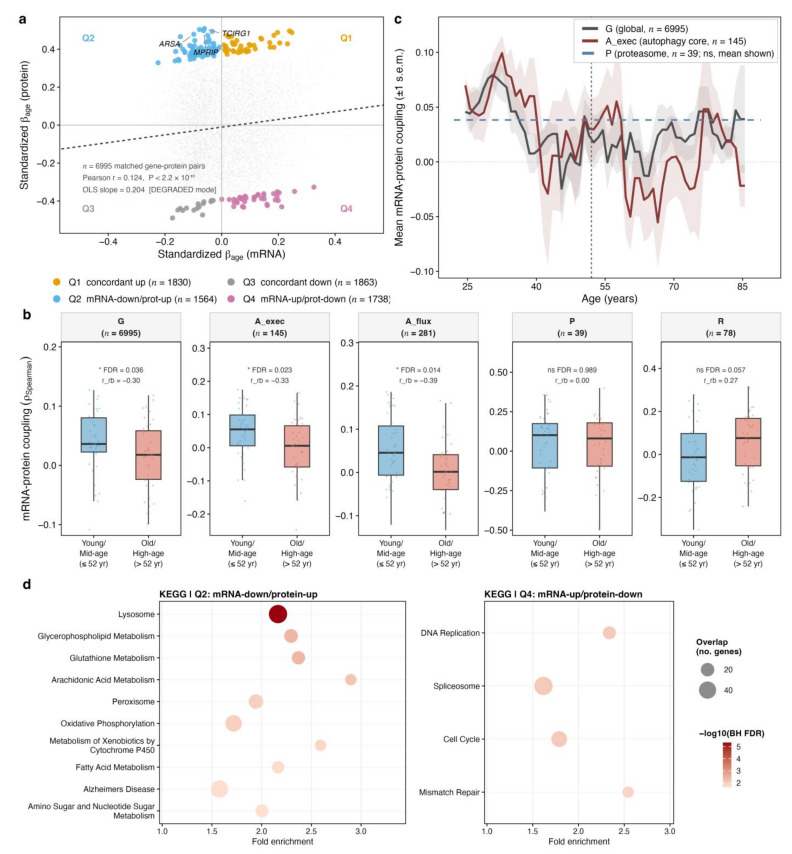
Pervasive mRNA–protein decoupling in ageing skin fibroblasts with pathway-selective coupling decline. (**a**) Standardised age-regression coefficients (*β*_age_, s.d. yr^−1^) for 6995 matched mRNA–protein pairs from 82 GESTALT donors (aged 22–89 years). Points with |*Z*_decoupling_| > 2 (*n* = 153) are coloured according to quadrant: orange for Q1, blue for Q2, dark grey for Q3, and magenta for Q4; all remaining points are shown in light grey. The black dashed line represents the ordinary least-squares (OLS) regression fit. Key statistics are annotated in the panel. Selected proteins with high decoupling scores are labelled. (**b**) Per-donor mRNA–protein coupling index (Spearman *ρ* across paired genes) in lower-age (≤52 years, *n* = 42) and higher-age (>52 years, *n* = 40) subgroups across five prespecified gene sets;subset definitions are provided in Table S1. Blue and salmon indicate the lower-age and higher-age groups, respectively. Boxes show the median and interquartile range (IQR); whiskers extend to 1.5 × IQR; dots represent individual donors. Benjamini–Hochberg (BH)-corrected two-sided Mann–Whitney U-test false discovery rate (FDR) values for 12 planned comparisons and rank-biserial correlation coefficients (r_rb_) are indicated for each subset. An asterisk (*) indicates FDR < 0.05, whereas ns indicates FDR ≥ 0.05. (**c**) Sliding-window mRNA–protein coupling calculated using 5-year windows with 1-year steps for three gene sets. The grey and dark-red solid lines represent the mean coupling for the global (G) and autophagy-core (A_exec_) gene sets, respectively, and the corresponding grey and dark-red shaded areas indicate ±1 standard error of the mean (s.e.m.). The blue horizontal dashed line represents the cohort-wide mean coupling for the proteasome (P) gene set, for which no significant age-dependent trend was detected (FDR = 0.989). The grey vertical dashed line marks the cohort median age of 52 years. (**d**) KEGG pathway over-representation for Q2 (protein-accumulating, *n* = 1564 genes) and Q4 (protein-depleting, *n* = 1738 genes) under the DEGRADED-mode interpretation, using 6957 Entrez-mappable genes as the background and a BH FDR threshold of <0.05; gene sets were obtained using msigdbr. Bubble area represents the number of overlapping genes, and fill colour represents −log_10_(BH FDR). GO Biological Process results are in Figure S1.

To quantify whether transcript and protein ageing trajectories remained aligned in primary skin fibroblasts, we analysed 6995 matched mRNA–protein features across 82 GESTALT donors aged 22–89 years. Global correspondence was weak: the Pearson correlation between matched mRNA and protein age-regression coefficients was *r* = 0.124 (*R*^2^ = 1.5%; Figure 1a). The residual-based decoupling score collapsed onto *β*_protein_ rankings (*r*_residual–protein_ = 0.992), satisfying the prespecified DEGRADED-mode criterion; under this criterion, the residual-based decoupling score reduces to a protein age-regression coefficient, and subsequent candidate identification reflects protein-layer ageing outliers rather than strict mRNA–protein decoupling targets. Nearly half of gene–protein pairs (47.2%; Q2 + Q4) showed discordant ageing trajectories. PERMANOVA on the sex-corrected matrices confirmed independent age-group separation in both layers (transcriptome *R*^2^ = 0.047, *p* = 0.038; proteome *R*^2^ = 0.056, *p* = 0.001; *n* = 82 donors; Euclidean distance; 999 permutations; Figure S2).

We next performed over-representation analysis (ORA) on the two discordant quadrants under DEGRADED-mode interpretation (Figure 1d and Figure S1). Among proteins that increased with age despite declining transcripts (Q2, *n* = 1564), KEGG Lysosome ranked first (BH FDR < 0.001), and GO Biological Process terms were dominated by lipid and small-molecule catabolic processes (Figure S1). Proteins depleted with age despite rising transcripts (Q4, *n* = 1738) were enriched for RNA splicing and DNA replication (GO-BP FDR = 2.3 × 10^−12^ for both; KEGG Spliceosome and DNA Replication, both FDR = 0.009; Figure 1d), consistent with proliferative quiescence of aged fibroblasts. The Q2 enrichment is consistent with protein-layer-dominant remodelling of the autophagy–lysosome axis as a principal feature of the discordant proteome.

To test whether this near-zero global coupling reflected an age-progressive process, we computed a per-donor Spearman coupling index and divided donors at the cohort median age (52 years) into lower-age (*n* = 42) and higher-age (*n* = 40) groups. Coupling declined in the global gene set (G, *n* = 6995 paired genes; two-sided Mann–Whitney *U* test, BH FDR = 0.036, *r*_rb_ = −0.30) and the effect was larger in the autophagy execution subset (A_exec_, *n* = 145; FDR = 0.023, *r*_rb_ = −0.33) and autophagy flux subset (A_flux_, *n* = 281; FDR = 0.014, *r*_rb_ = −0.39; Figure 1b). The proteasomal subset showed no decline (P, *n* = 39; FDR = 0.989), indicating pathway-selective rather than uniform deterioration. Sliding-window trajectories placed the onset of the autophagy–proteasome divergence near mid-life Figure 1c). Stratified bootstrap testing of the A_exec_–global differential was directionally consistent but fell short of significance (Δρ = 0.031, *P*_boot_ = 0.055), setting the boundary for the autophagy-selective coupling result before the module-level tests below.

### 2.2. A Dual-Track Candidate Signature Converges on the Proteome-Layer Ageing Signal

**Figure 2:**
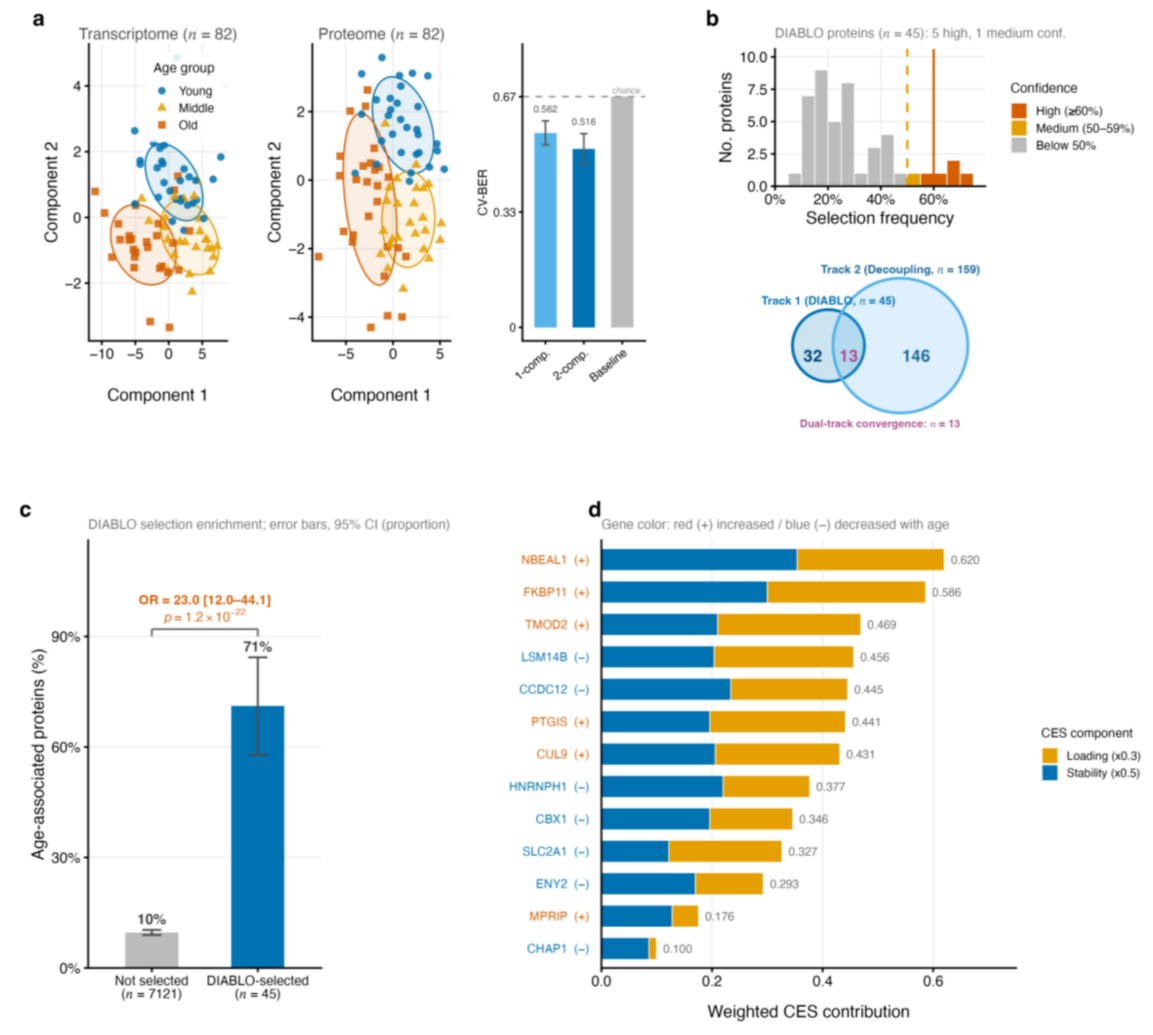
A dual-track candidate signature converges on the proteome-layer ageing signal. (**a**) DIABLO sample scores on discriminant components 1 and 2 for the transcriptomic (left) and proteomic (right) blocks (*n* = 82 donors, coloured by age tertile; shaded ellipses, 68% confidence regions). Inset: cross-validated balanced error rate (CV-BER; 5-fold × 50 repeats, centroid-distance classifier) for 1-component, 2-component and random-baseline models; error bars, ±1 s.d.; dashed line, random-chance BER = 0.667. (**b**) Upper: cross-validation selection frequency for the 45 DIABLO-selected proteins (Track 1 proteomic block). Vertical lines mark high-confidence (≥60%, solid) and medium-confidence (50–59%, dashed) stability thresholds. Lower: Euler diagram of Track 1 proteins (DIABLO proteomic block, *n* = 45) and Track 2 (protein-layer age-outlier screen, *n* = 159). Circle areas are proportional to set size. Numbers show protein counts per zone; 13 proteins appear in both tracks (dual-track convergence candidates). Track 1 additionally contains 76 transcriptomic features, which are not included in the Euler diagram because the diagram compares protein sets and Track 2 comprises proteins only. (**c**) Proportion of age-associated proteins (independent linear regression, BH FDR < 0.05) within DIABLO-selected (*n* = 45) versus not-selected (*n* = 7121) proteins. Error bars, 95% confidence intervals (Wald method). OR, odds ratio with 95% CI (Woolf method on log scale); *p*, two-sided Fisher’s exact test. (**d**) Composite evidence scores (CES) for the 13 dual-track convergence candidates, decomposed into selection stability (×0.5, blue) and component loading (×0.3, amber). The decoupling term (×0.2) is set to zero for all candidates under DEGRADED mode. Gene labels: red (+), increased with age; blue (−), decreased with age. CES values are shown at the right of each bar.

We next asked whether supervised multi-omics integration could nominate molecular candidates associated with the proteome-layer ageing divergence. A sparse DIABLO model with two discriminant components and a null inter-block design achieved a cross-validated balanced error rate (CV-BER) of 0.516 ± 0.044 (5-fold × 50 repeats), a 22.5% improvement over the three-class random baseline of 0.667 (Figure 2a). Age-label permutation testing (n = 100 permutations; 5-fold cross-validation) confirmed that the observed CV-BER (0.516) was significantly lower than the permutation null distribution (permutation *p* = 0.020; Figure S3e), ruling out chance as an explanation for the above-baseline classification improvement. The elevated Middle-class error (58.4% versus 49.3% for Young and 47.2% for Old) was consistent with the biological continuity of the midlife transition (Figure S3a–d).

The union of DIABLO-selected features across both components constituted Track 1 (121 features: 76 transcripts and 45 proteins). Of the 45 selected proteins, 32 (71%) were independently confirmed as age-associated by single-omics linear regression (FDR < 0.05; Fisher’s exact test OR = 23.0, 95% CI 12.0–44.1, *p* = 1.2 × 10^−22^; Figure 2c), arguing against selection dominated by age-irrelevant discriminative features. Track 2 was derived from the protein-layer age-outlier screen (operating under DEGRADED mode, in which decoupling scores reflect proteomic age association; see Materials and Methods): 153 proteins met the Tier A criterion (|*Z*_decoupling_| > 2 jointly with proteomic FDR < 0.05), with 6 additional features from the Tier C adaptive threshold, giving 159 Track 2 proteins in total. The intersection of Tracks 1 and 2 yielded 13 dual-track convergence candidates, each supported by both supervised multi-omics discrimination and proteome-layer age association (Figure 2b).

Ranking these 13 proteins by composite evidence score (CES), which weights selection stability (×0.5) and component loading (×0.3), placed NBEAL1 (CES = 0.620; increased with age), FKBP11 (0.586; increased), TMOD2 (0.469; increased) and LSM14B (0.456; decreased) at the top positions (Figure 2d). Under DEGRADED mode (protein-layer age-outlier interpretation) the decoupling term was set to zero, so CES reflected stability and loading only. CES rankings were stable across two alternative weighting configurations (all pairwise Spearman *ρ* ≥ 0.998; Jaccard top 25 ≥ 0.85; Table S3). Prostacyclin synthase (PTGIS; CES = 0.441) was the sole candidate captured at the protein level by both DIABLO discrimination (Component 2) and the proteome-layer outlier screen (|*Z*_decoupling_| = 2.62). The complete 267-feature candidate pool and CES decomposition are provided in Table S2.

### 2.3. Autophagy–Lysosome Proteins Mark Transcriptome-Uncoupled Proteome Remodelling with Age

**Figure 3:**
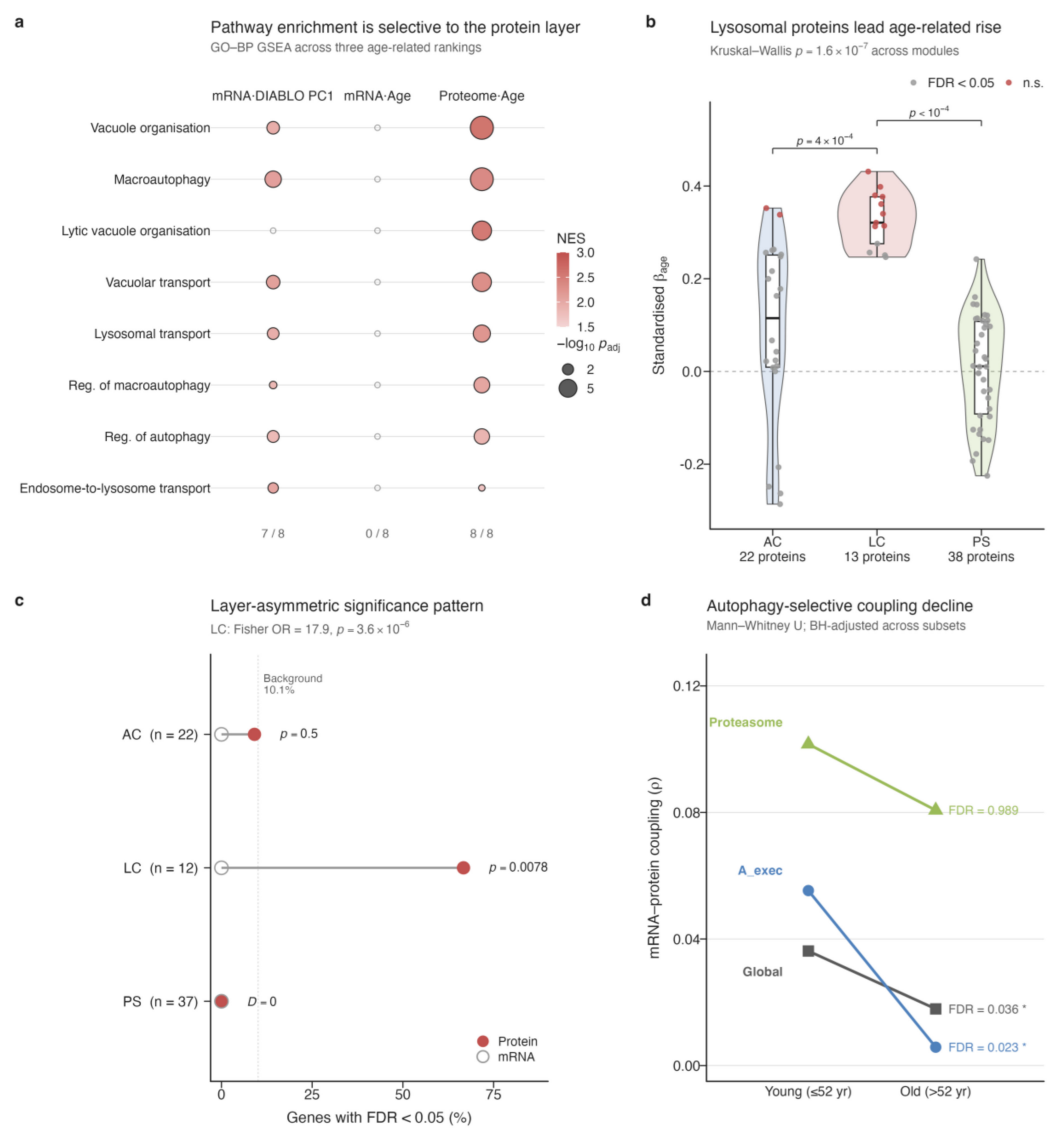
Autophagy–lysosome proteins mark transcriptome-uncoupled proteome remodelling with age. (**a**) GO Biological Process GSEA of eight prespecified autophagy–lysosome terms across three age-related feature rankings: mRNA ranked by Pearson correlation with DIABLO component 1 scores, mRNA ranked by univariate linear age association and proteins ranked by sex-adjusted age-regression *t* statistic. Bubble area represents −log_10_ of the Benjamini–Hochberg (BH)-adjusted *p* value, and fill represents the normalised enrichment score (NES). Numbers below each column indicate the count of terms reaching FDR < 0.05 out of eight tested. Full GSEA results are in Figure S4. (**b**) Standardised protein age-regression coefficients (*β*_age_) for the three prespecified proteostasis modules: autophagy core (AC, *n* = 22 proteins), lysosomal degradative-capacity (LC, *n* = 13) and proteasomal (PS, *n* = 38). Violin plots show density; boxes indicate the median and interquartile range (IQR); dots represent individual proteins. Filled dots indicate BH FDR < 0.05, whereas open dots indicate FDR ≥ 0.05. Kruskal–Wallis omnibus *p* and Dunn post hoc BH-adjusted pairwise *p* values are annotated. (**c**) Layer-asymmetric significance pattern across the three modules. For each module, the percentage of member genes reaching BH FDR < 0.05 is shown separately for the protein layer (filled circles) and the transcript layer (open circles). The dashed vertical line indicates the proteome-wide background rate of 10.1%. Two-sided exact McNemar *p* values test whether the discordant counts (*n*_10_ vs. *n*_01_) differs from symmetry. LC enrichment relative to background: Fisher OR = 17.9, *p* = 3.6 × 10^−6^. (**d**) Mean per-donor mRNA–protein coupling (Spearman *ρ*) in lower-age (≤52 years) and higher-age (>52 years) subgroups for the autophagy execution (A_exec_), global (G) and proteasomal (P) gene sets, presented as a slope summary to facilitate comparison with the module-level results in panels **b** and **c**. BH-adjusted two-sided Mann–Whitney *U* test FDR values are shown; *, FDR < 0.05.

We next asked whether the autophagy–proteasome divergence detected by global coupling analysis extended to defined proteostasis modules. GSEA was applied to three age-related feature rankings: mRNA ranked by correlation with DIABLO component 1, mRNA ranked by linear age association and proteins ranked by the age-regression *t* statistic, using GO Biological Process, Hallmark and Reactome collections (Figure 3a). The two omics layers separated sharply. Linear mRNA–age GSEA returned only four GO-BP gene sets at FDR < 0.05, none autophagy- or lysosome-related. The proteome–age ranking returned 340 GO-BP gene sets at FDR < 0.05, headed in the positive direction by vacuole organisation (NES = 2.60, BH-adjusted *p* = 1.0 × 10^−9^) and macroautophagy (NES = 2.36, *p* = 1.6 × 10^−9^), with RNA splicing leading the negative direction (NES = −3.23, *p* = 3.7 × 10^−32^; Figure S4). The DIABLO component 1 mRNA ranking recovered seven of the eight autophagy–lysosome terms in Figure 3a (NES range 1.81–2.12), indicating that supervised integration captured a coordinated transcriptional autophagy programme that univariate mRNA–age regression did not detect. A Fisher’s exact test of the dual-track candidate set against the autophagy reference gene set (GO:0006914 ∪ GO:0016236) returned no enrichment (protein OR = 1.00, *p* = 0.60; mRNA *p* = 1.00), indicating that the pathway-level signal was distributed more broadly than the 267 integration-nominated features.

To localise the protein-layer enrichment, we tested three prespecified proteostasis modules (Figure 3b). Standardised age coefficients differed across modules (Kruskal–Wallis H = 31.25, df = 2, *p* = 1.6 × 10^−7^). The LC module dominated: nine out of 13 LC proteins reached FDR < 0.05, all 13 carried positive age coefficients (median standardised *β* = 0.32; sign test *p* = 4.9 × 10^−4^), and the LC set was 17.9-fold over-represented relative to the proteome-wide rate of 10.1% (one-sided Fisher’s exact test, *p* = 3.6 × 10^−6^). Dunn post hoc comparisons with BH correction separated LC from AC (adjusted *p* = 4.0 × 10^−4^) and PS (adjusted *p* < 1.0 × 10^−4^). The three dual-track candidates mapping to any module, CTSA, CTSB and ATP6V0D1, all fell within LC. The AC module was directionally consistent (18 of 22 positive, sign test *p* = 4.3 × 10^−3^) but individually weaker: only ATG9A (*β* = 0.34, Q2 quadrant) and ATG5 (*β* = 0.35, Q1) crossed FDR < 0.05, linking upstream autophagosome machinery with downstream lysosomal hydrolases. The PS module contained no proteins at FDR < 0.05 (mean *β* = 0.006, 20 of 37 positive, sign test *p* = 0.74), consistent with maintained proteasome stoichiometry across the adult lifespan.

Paired McNemar analysis tested whether the LC and AC effects were layer-asymmetric (Figure 3c). Across all 6995 paired genes no transcript reached FDR < 0.05 after BH correction, so every discordant pair was protein-only. Within LC_paired_ (*n* = 12), eight proteins met FDR < 0.05 against zero transcripts (D = 8; two-sided exact McNemar *p* = 0.0078). AC_paired_ yielded D = 2 (*p* = 0.50) at the strict threshold; relaxing the protein layer to FDR < 0.10 raised D to 6 (*p* = 0.031), with the same direction of asymmetry. PS_paired_ had D = 0, with neither layer carrying an age-associated signal. This module-resolved asymmetry was consistent with the coupling-index analysis (Figure 3d): A_exec_ coupling declined with age (Δρ = 0.049, FDR = 0.023), the global set declined more weakly (Δρ = 0.018, FDR = 0.036) and the proteasomal subset did not decline (FDR = 0.989). Whether this protein-layer lysosomal signal emerged without pathway supervision was tested next.

### 2.4. A Lysosomal Co-Expression Module Overlaps the Multi-Omics Ageing Signature

**Figure 4:**
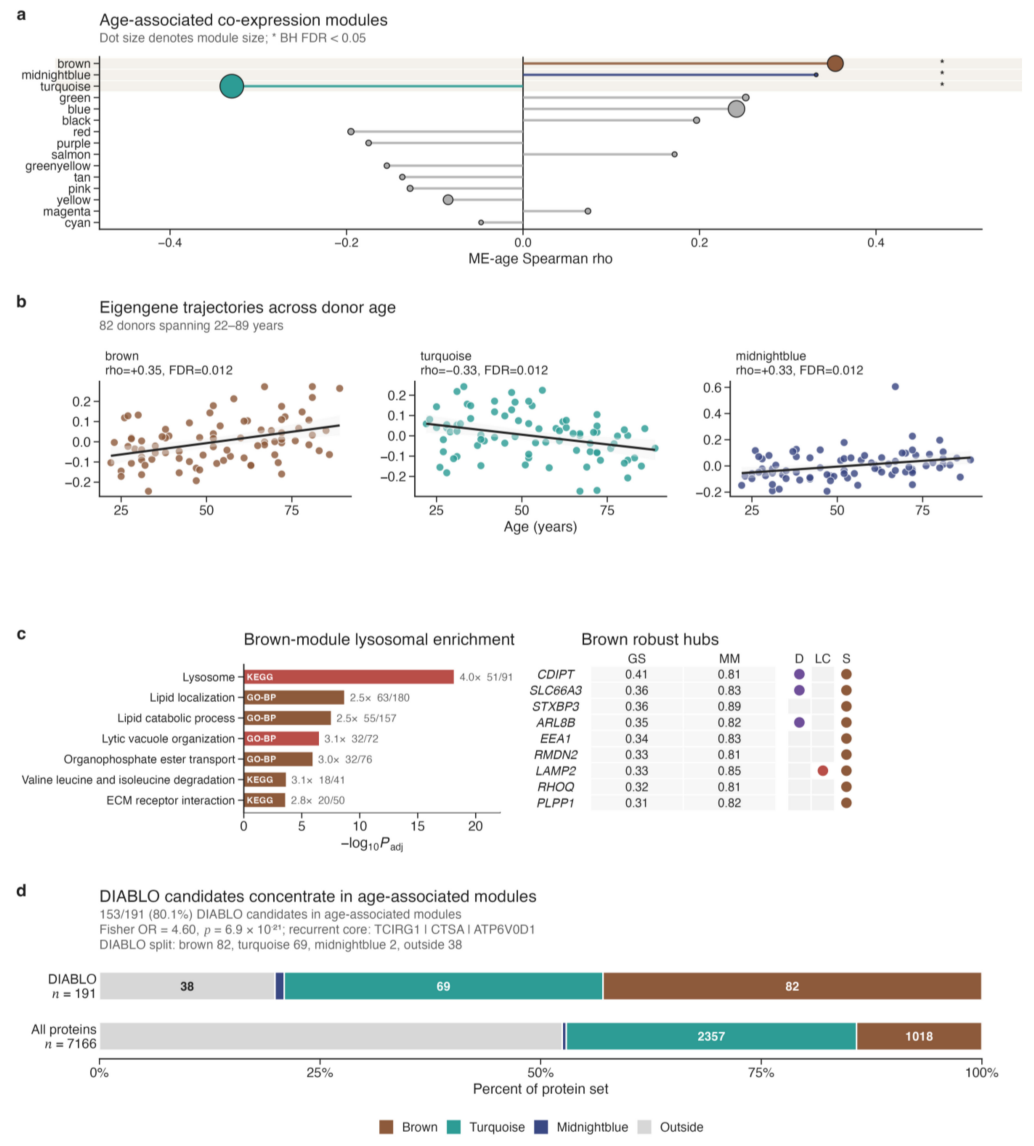
Unsupervised protein co-expression analysis identifies an age-associated lysosomal module overlapping the multi-omics signature. (**a**) Module eigengene (ME) correlation with chronological age for the 15 non-grey WGCNA modules detected from 7166 quantified proteins across 82 GESTALT fibroblast donors. Points show Spearman ρ between each ME and age; point size denotes module size, and point colours correspond to the WGCNA module colours. Shaded rows indicate modules significant after BH correction across modules (FDR < 0.05). (**b**) ME trajectories across donor age for the three age-associated modules. Points represent individual donors, and point colours correspond to the respective WGCNA module colours; lines represent linear fits for visualisation. Spearman ρ and BH-adjusted FDR are shown in each facet. (**c**) Left: over-representation analysis of the brown module against the quantified protein background; bar length represents −log_10_(BH-adjusted p), with labels indicating fold enrichment and overlap count. Red bars highlight lysosomal terms, whereas brown bars indicate the remaining enriched terms. Right: nine robust hubs in the brown module meeting MM ≥ 0.80 and GS_age ≥ 0.30, all retained after GT-002 sensitivity analysis. Purple, red, and brown dots indicate DIABLO candidate status (D), membership in the predefined lysosomal degradative-capacity panel (LC), and retention after GT-002 removal (S), respectively. (**d**) Distribution of DIABLO multi-omics protein candidates (*n* = 191) and all quantified proteins (*n* = 7166) across brown, turquoise, midnightblue and outside (non-age-associated) modules. Brown, turquoise, and midnightblue segments represent the corresponding age-associated WGCNA modules, whereas grey segments represent proteins outside these modules. Fisher’s exact test (one-sided) used all quantified proteins as background; OR and *p* are annotated. Recurrent core proteins (TCIRG1, CTSA, and ATP6V0D1) are noted.

To test whether the autophagy–lysosome signal identified by supervised integration and module-targeted regression also emerged without pathway annotation, we performed WGCNA on 7166 quantified proteins across 82 donors. The signed hybrid network (β = 6; scale-free R^2^ = 0.92) yielded 15 non-grey modules ranging from 33 to 2357 proteins (Figure S5a,b). Three modules were age-associated after BH correction across the 15 non-grey modules: brown (1018 proteins; Spearman *ρ* = +0.354, FDR = 0.012), turquoise (2357 proteins; *ρ* = −0.330, FDR = 0.012) and midnightblue (33 proteins; *ρ* = +0.332, FDR = 0.012; Figure 4a,b; Table S4). Leave-one-out removal of the strongest outlier (GT-002) preserved brown and turquoise at FDR < 0.05; midnightblue weakened to FDR = 0.083 and was treated as exploratory (Figure S5c,d).

The brown module was the primary biological focus. KEGG_LYSOSOME was the strongest enriched pathway in the entire network analysis (adjusted *p* = 7.7 × 10^−19^, fold-enrichment = 4.0, 51 of 91 pathway proteins captured; Figure 4c; Table S5), followed by GO-BP terms for lipid localisation (adjusted *p* = 2.2 × 10^−9^), lytic vacuole organisation (adjusted *p* = 3.4 × 10^−7^) and macroautophagy (adjusted *p* = 2.0 × 10^−2^). Brown also carried a structured V-ATPase signal: six subunits mapped to brown (V0 sector: TCIRG1, ATP6V0A2, ATP6V0A4; V1 sector: ATP6V1C1, ATP6V1D, ATP6V1F), whereas the midnightblue module carried five V1-sector subunits exclusively (ATP6V1A, ATP6V1B2, ATP6V1E1, ATP6V1G1, ATP6V1H), suggesting that V-ATPase components partition across two age-associated co-expression contexts. Nine brown proteins met the robust hub criterion (MM ≥ 0.80, GS_age_ ≥ 0.30) and were retained after GT-002 removal: CDIPT, SLC66A3, STXBP3, ARL8B, EEA1, RMDN2, LAMP2, RHOQ and PLPP1 (Figure 4c; Table S6). The turquoise module showed the opposite age direction: its biology was governed by RNA splicing (GO-BP adjusted *p* = 1.4 × 10^−81^) and DNA replication (KEGG adjusted *p* = 4.3 × 10^−12^), and its hubs included MCM2/3/6, RFC1/4, MSH6 and HNRNPH1, recapitulating the downregulated proteomic arm from genome-wide GSEA.

We finally tested whether the supervised and unsupervised approaches converged on the same protein set. Of the 191 protein candidates from the dual-track pool, 153 (80.1%) fell within one of the three age-associated modules (brown 82, turquoise 69, midnightblue 2; one-sided Fisher’s exact OR = 4.60, *p* = 6.9 × 10^−21^; Figure 4d). Three proteins appeared across all four complementary analyses (DIABLO dual-track selection, predefined LC module, McNemar protein-layer-only signal and brown co-expression module): TCIRG1, CTSA and ATP6V0D1. The WGCNA therefore provided an unsupervised complement to the layer-asymmetry and dual-track results; noting that all approaches draw on the same GESTALT proteomic dataset, their convergence on the autophagy–lysosome axis represents internally consistent, complementary evidence rather than independent replication.

### 2.5. External GTEx Transcriptomes Contextualise the AC/LC Signal to the Protein Layer

We next asked whether the proteomic AC/LC > PS asymmetry in fibroblasts reflected transcriptional regulation or a post-transcriptional event. GTEx v8 provided an external tissue-level transcriptomic reference: Skin—Not Sun Exposed (Suprapubic; *n* = 639) was anatomically closest to the GESTALT biopsy site, and Skin—Sun Exposed (Lower Leg; *n* = 768) assessed photoageing-related effects. A transcriptional-cascade model predicted that the AC/LC > PS asymmetry would recapitulate at the mRNA layer; a post-transcriptional model predicted a weak or directionally inconsistent signal. We note that GTEx whole-skin samples contain multiple cell types, use age-bin encoding rather than continuous age, and are subject to post-mortem variables; this external comparison therefore provides tissue-level context rather than a direct validation of the fibroblast findings.

**Figure 5:**
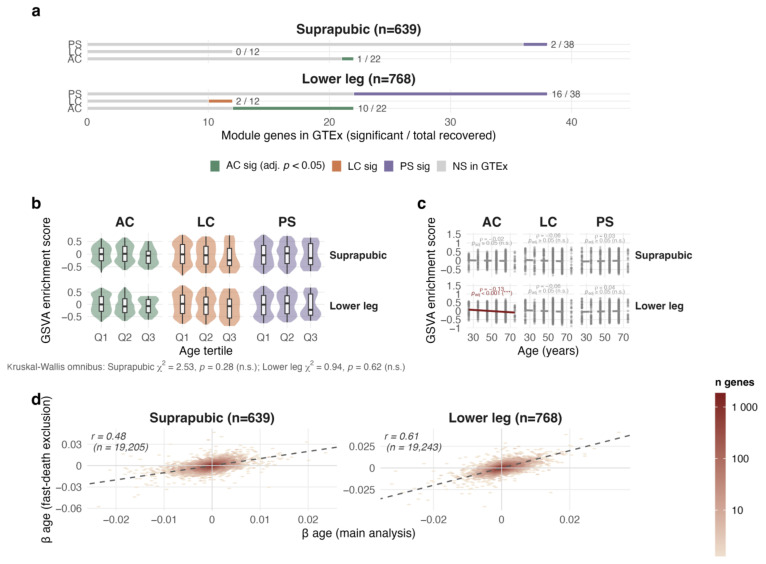
External GTEx transcriptomes contextualise the AC/LC signal to the protein layer. (**a**) Number of module genes reaching BH-adjusted *p* < 0.05 for age association in GTEx v8 Skin—Not Sun Exposed (Suprapubic, *n* = 639 donors) and Skin—Sun Exposed (Lower Leg, *n* = 768). Coloured segments indicate significant genes in each module: green for AC, orange for LC, and purple for PS; grey segments indicate non-significant genes.Fractions denote significant/total recovered genes per module. The GESTALT fibroblast proteome module sizes (AC = 22, LC = 13, PS = 38) were mapped at the HGNC symbol level; 72 of 73 members were recovered in both tissues. (**b**) GSVA sample-level enrichment scores for the AC, LC, and PS modules across GTEx age tertiles (Q1–Q3) in Suprapubic (upper) and Lower Leg (lower). Boxes indicate the median and IQR, and whiskers extend to 1.5 × IQR.. Kruskal–Wallis omnibus *p* values (transcript-level *β*_age_ across modules) are annotated below the panel. (**c**) GSVA enrichment scores versus continuous donor age (midpoint-encoded decade bins) for each module in Suprapubic (upper) and Lower Leg (lower). Spearman *ρ* and BH-adjusted significance are shown per facet (n.s., adjusted *p* ≥ 0.05; ***, adjusted *p* < 0.001). Lines represent linear fits for visualisation. Solid lines indicate BH-adjusted *p* < 0.05, whereas dashed lines indicate BH-adjusted *p* ≥ 0.05. (**d**) Gene-level age-regression coefficients (*β*_age_) from the full-cohort analysis (*x*-axis) versus the prespecified fast-death subset (Hardy scale 1 or 2; *y*-axis) in Suprapubic (*n* = 189 donors) and Lower Leg (*n* = 236). The grey dashed diagonal line represents the identity line (*y* = *x*). Colour intensity represents local gene density.

Module-level transcript age effects did not reproduce the proteomic asymmetry. Kruskal–Wallis tests of transcript *β*_age_ across AC, LC and PS returned *p* = 0.28 in Suprapubic and *p* = 0.62 in Lower Leg, compared with *p* = 1.6 × 10^−7^ at the protein layer. At the single-gene level, Suprapubic carried little signal (AC 1/22, LC 0/12, PS 2/38 at BH *p* < 0.05; Figure 5a). Lower Leg recovered more hits (AC 10/22, LC 2/12, PS 16/38), but the dominant PS contribution ran counter to the quiescent PS proteome (0/37 at FDR < 0.05), a pattern more consistent with UV-induced cell-stress transcription than chronological ageing.

GSVA enrichment scores localised the transcript-layer boundary most clearly to LC (Figure 5b,c). In Suprapubic, no module carried an age-correlated signal (AC *ρ* = −0.025, LC *ρ* = −0.059, PS *ρ* = +0.034; all BH *p* > 0.40). LC provided the sharpest cross-layer contrast: nine of 13 proteins reached FDR < 0.05 with a median standardised *β* of 0.32 in the GESTALT proteome, yet the same gene set was flat at the transcript layer in both tissues (Suprapubic *ρ* = −0.059; Lower Leg *ρ* = −0.062). The only significant association was Lower Leg AC (*ρ* = −0.130, BH *p* = 8.65 × 10^−4^; Figure 5c). AC transcripts declined with age in sun-exposed skin, whereas AC proteins increased in the fibroblast proteome (18/22 positive, sign test *p* = 4.3 × 10^−3^), matching the Q2 protein-up/transcript-down configuration from the global decoupling analysis (Figure 1a,d). A fast-death sensitivity analysis (Hardy 1 or 2; Suprapubic *n* = 189, Lower Leg *n* = 236) preserved gene-level directionality in both tissues (Pearson *r* = 0.48 and 0.61, respectively; Figure 5d). The external GTEx transcriptomic comparison therefore showed no module-level AC/LC > PS asymmetry at the transcript layer, consistent with protein-layer-dominant remodelling of the lysosomal degradative axis.

### 2.6. Network Medicine Nominates Autophagy–Lysosome-Biassed Nutraceuticals from the Ageing Proteostasis Module

**Figure 6:**
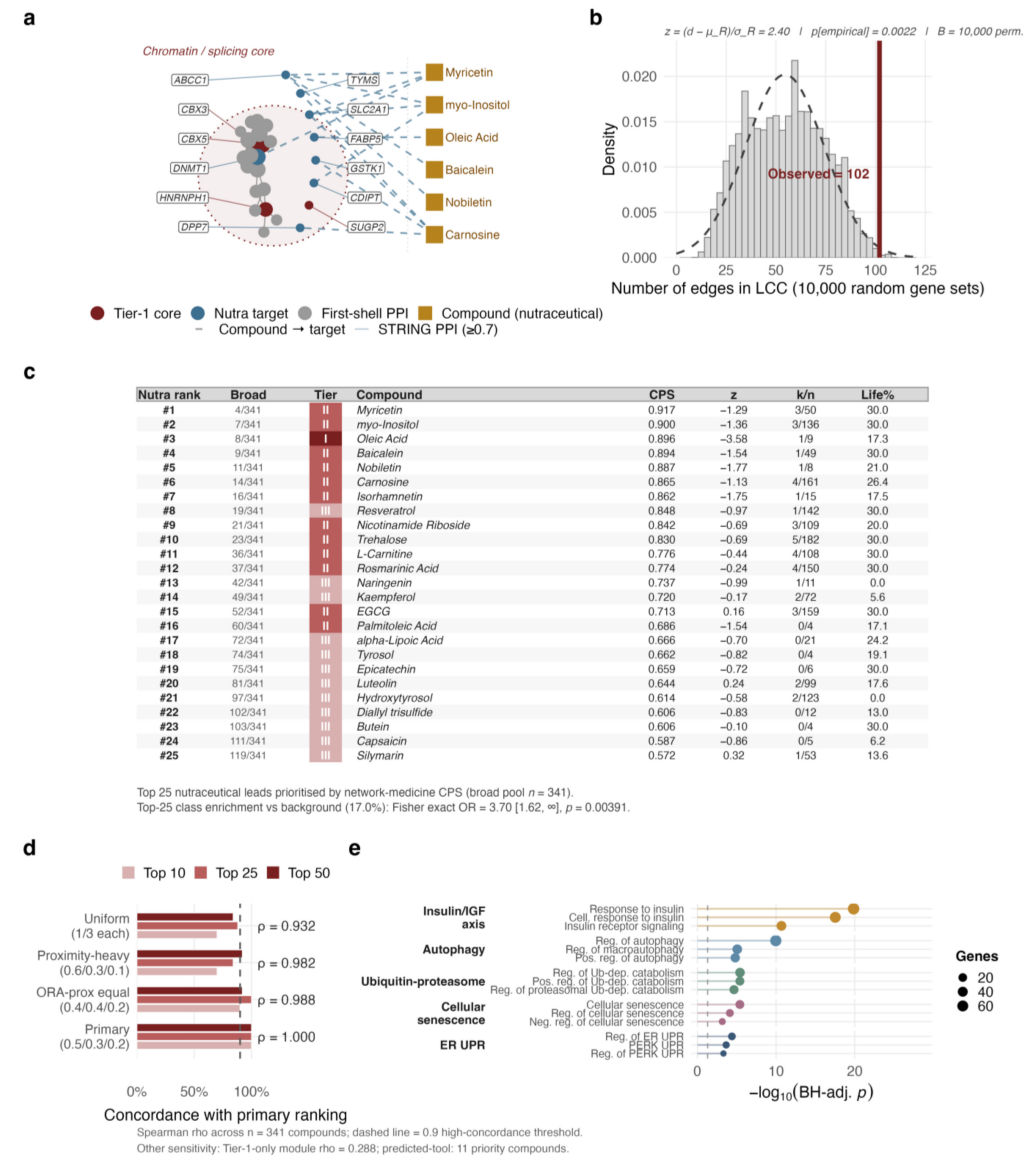
Network medicine prioritisation nominates dietary nutraceuticals for the ageing proteostasis module. (**a**) Subgraph of the 244-protein ageing target module projected onto the STRING v12.0 high-confidence interactome. Red nodes indicate core-module (Tier-1) hubs, grey nodes indicate first-shell protein–protein interaction (PPI) partners, blue nodes indicate nutraceutical target proteins within the module, and orange squares indicate top-ranking nutraceutical compounds. Blue dashed edges indicate compound-to-target links, solid grey edges indicate STRING PPIs with a combined score ≥ 0.700, and red dotted edges indicate interactions within the labelled chromatin/splicing core. (**b**) Largest connected component (LCC) significance test. The observed module LCC size of 102 edges, indicated by the red vertical line, is compared with the null distribution shown by the grey histogram and black dashed density curve. The null distribution was generated from 10,000 degree-matched random gene sets, each containing 244 proteins, sampled from the 15,882-node STRING-LCC background. The *z*-score, empirical *p* value, and permutation count are annotated. (**c**) Top 25 nutraceutical leads ranked by composite prioritisation score (CPS) from the full compound pool (*n* = 341). The hash symbol (#) denotes the nutraceutical rank position.Columns show nutraceutical rank, broad rank among all compounds, CPS tier (I, II, or III), compound name, CPS value, network-proximity *z*-score, direct target overlap with the module (*k/n*), and the maximum lifespan extension reported in DrugAge (Life%). Dark, medium, and light red shading in the Tier column indicate Tiers I, II, and III, respectively.Top 25 class enrichment versus the 17.0% nutraceutical background: Fisher’s exact OR = 3.70 [1.62, ∞], *p* = 0.0039. (**d**) CPS weight-sensitivity analysis. Light, medium, and dark red horizontal bars show the proportions of the top 10, top 25, and top 50 compounds, respectively, that are shared with the primary weighting scheme (0.5/0.3/0.2) under three alternative configurations. Spearman ρ for the full-pool CPS ranks is annotated for each scheme. The grey vertical dashed line indicates the high-concordance threshold of ρ = 0.9. Additional sensitivity notes are listed below the panel. (**e**) Pathway over-representation analysis of the Level I + II compound-target union (54 compounds and 1191 unique targets) against the STRING-LCC background (*n* = 15,882). Gene Ontology (GO) Biological Process and Kyoto Encyclopedia of Genes and Genomes (KEGG) terms are grouped into five prespecified hallmark-of-ageing pathway families. Gold, blue, green, mauve, and dark-blue bubbles denote the insulin/IGF axis, autophagy, ubiquitin–proteasome, cellular senescence, and endoplasmic reticulum unfolded protein response (ER UPR) families, respectively. Bubble area represents the number of overlapping genes, and the horizontal position represents −log_10_(BH-adjusted *p*).

We next asked whether the 244-protein ageing target module occupied a non-random interactome region that could be queried for dietary nutraceuticals. Of the 244 proteins, 228 (93.4%) mapped to the STRING v12.0 LCC (15,882 nodes, 473,424 edges) and formed a connected subgraph significantly exceeding the degree-matched null (*z* = +2.40, empirical *p* = 0.0022; 10,000 permutations; Figure 6a,b). The module interior joined chromatin/splicing regulators (CBX3, CBX5, HNRNPH1, SUGP2 and first-shell partners) with lysosomal and V-ATPase components, bridging the turquoise and brown co-expression arms into a single network neighbourhood.

From a compound library of 341 candidates with 3–200 mapped targets (median 12), 58 (17.0%) were annotated as nutraceuticals. We note that compound–target databases are incomplete and biassed toward extensively studied molecules; network proximity scores therefore reflect current annotation coverage and represent computational prioritisation signals rather than evidence of biological activity. Network proximity recovered interactome topology that direct overlap testing missed (16 compounds at *z* ≤ −2; 125 at *z* ≤ −0.15). Ranking by composite prioritisation score (CPS) produced nutraceutical enrichment in the top 25 list (10/25 nutraceuticals, 40.0%; Fisher’s exact OR = 3.70, *p* = 0.0039 against the 17.0% background; Figure 6c; Table S7). The top 25 leads spanned flavonoids (myricetin, baicalein, kaempferol, EGCG, luteolin and five others), polyphenols (resveratrol, hydroxytyrosol, tyrosol), unsaturated fatty acids (oleic acid, palmitoleic acid), and additional dietary classes including trehalose, carnosine, nicotinamide riboside and L-carnitine. CPS rankings were stable across three alternative weighting schemes (all Spearman *ρ* ≥ 0.93; top 25 overlap ≥ 84%; Figure 6d). Pathway over-representation of Level I + II compound targets (54 compounds, 1191 targets) recovered five hallmark-of-ageing families: the insulin/IGF axis (response to insulin, BH *p* = 1.4 × 10^−20^), autophagy regulation (*p* = 1.1 × 10^−10^), ubiquitin–proteasome (*p* = 3.8 × 10^−6^), cellular senescence (*p* = 4.0 × 10^−6^) and ER UPR (*p* = 4.1 × 10^−5^; Figure 6e).

**Figure 7:**
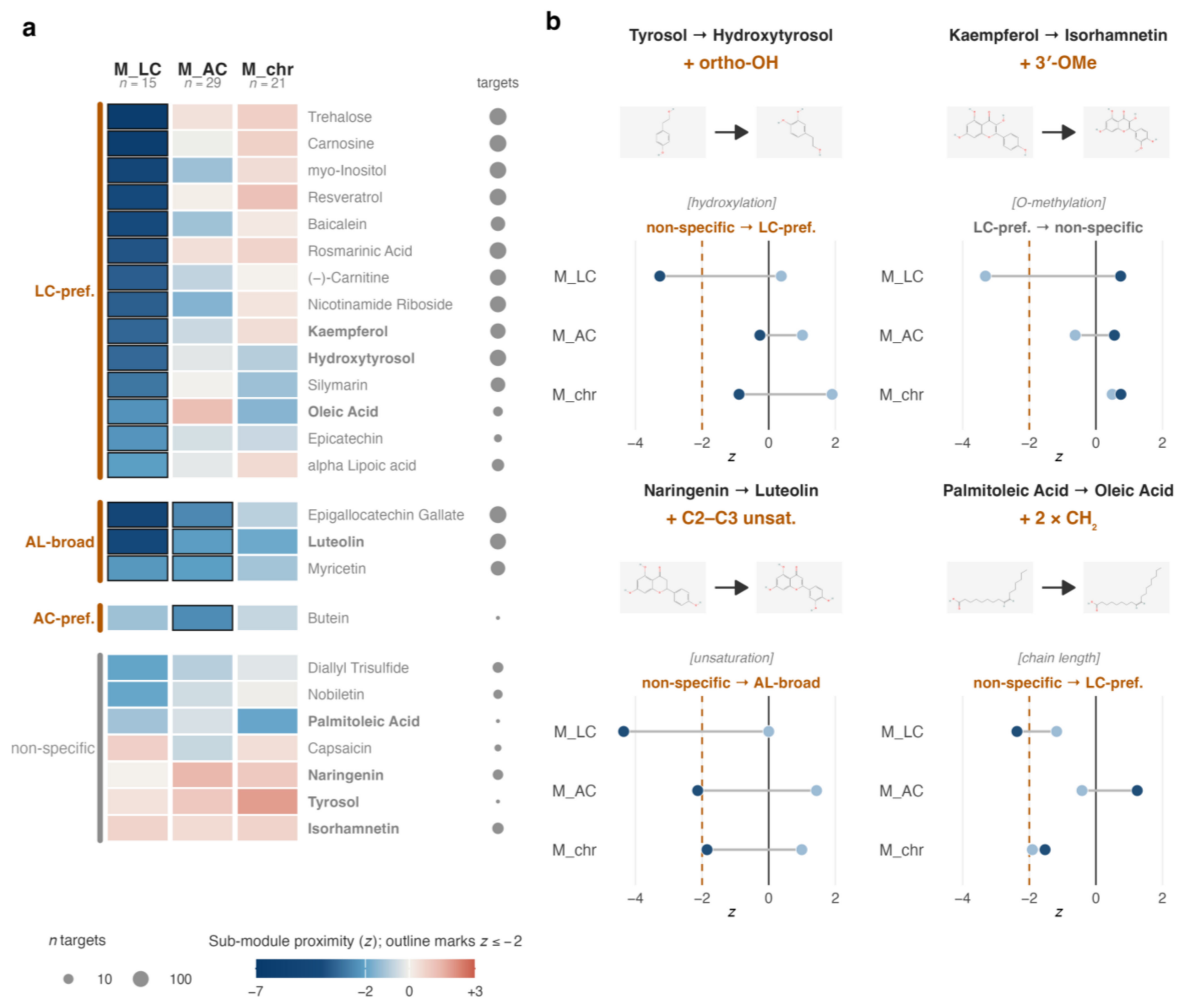
Sub-module-resolved network proximity identifies autophagy–lysosome-biassed nutraceutical niches. (**a**) Sub-module proximity *z*-scores for the top 25 CPS-prioritised nutraceuticals across three prespecified regions of the 244-protein ageing proteostasis module: M_LC_, lysosomal degradative-capacity sub-module (*n* = 15 proteins); M_AC_, autophagy core sub-module (*n* = 29 proteins); and M_chr_, chromatin/splicing sub-module plus first-shell PPI neighbours (*n* = 21 proteins). Compounds are grouped by assigned topological niche and ordered within each group by the most negative sub-module *z*-score. Black outlines mark cells meeting the canonical network-proximity threshold (*z* ≤ −2). Cell fill colour encodes the sub-module proximity *z*-score, ranging from dark blue for strongly negative values through near-white at zero to red for positive values.Orange side labels denote the autophagy–lysosome-targeting set (LC-preferential, autophagy–lysosome-broad and AC-preferential; 18 of 25 compounds, 72%). Grey dot area indicates the number of curated targets for each compound. (**b**) Intra-family structure-to-niche contrasts for four chemically related compound pairs selected a priori to represent distinct structural modifications: phenolic hydroxylation (tyrosol → hydroxytyrosol), 3′-O-methylation (kaempferol → isorhamnetin), C2–C3 unsaturation (naringenin → luteolin) and fatty-acid chain elongation (palmitoleic acid → oleic acid). Two-dimensional chemical structures are shown with the introduced feature highlighted in orange. Black arrows between each pair of structures indicate the direction from the parent compound to the structurally modified compound. Horizontal dumbbells show the per-sub-module proximity shift from the parent compound (light dot) to the modified compound, indicated by a light-blue dot, to the modified compound, indicated by a dark-blue dot; grey horizontal segments connect the paired values. The orange dashed vertical line marks the canonical proximity threshold of *z* = −2, whereas the black solid vertical line marks *z* = 0. Three of four contrasts moved the niche into the autophagy–lysosome-targeting set; one (3′-O-methylation) removed it.

To resolve whether the whole-module signal localised to the autophagy–lysosome arm, we computed closest-distance proximity of each top 25 nutraceutical to three sub-modules: M_LC_ (15 lysosomal degradative-capacity proteins), M_AC_ (29 autophagy core proteins) and M_chromatin_ (21 chromatin/splicing proteins). Fourteen compounds crossed *z* ≤ −2 only at M_LC_ (LC-preferential), led by trehalose (*z*_LC_ = −6.44) and carnosine (*z*_LC_ = −6.30; Figure 7a; Table S8). Three compounds (EGCG, luteolin, myricetin) reached significance at both M_LC_ and M_AC_ (autophagy–lysosome-broad). Butein was the sole AC-preferential compound, though its small target set (|*T*| = 4) placed this call under the prespecified low-power caveat. Seven compounds were non-specific; none preferentially targeted M_chromatin_. Pooling the autophagy–lysosome niches, 18 of 25 (72%) converged on the autophagy–lysosome arm rather than the chromatin/splicing arm.

Four prespecified matched-pair contrasts tested whether niche assignment was sensitive to single chemical modifications (Figure 7b). Adding an ortho-hydroxyl shifted tyrosol (non-specific; *z*_LC_ = +0.38) to hydroxytyrosol (LC-preferential; *z*_LC_ = −3.27). Introducing C2–C3 unsaturation shifted naringenin (non-specific) to luteolin (autophagy–lysosome-broad; *z*_LC_ = −4.36). Extending the fatty-acid chain by two methylenes shifted palmitoleic acid (non-specific) to oleic acid (LC-preferential; *z*_LC_ = −2.37). In a negative case, 3′-O-methylation shifted kaempferol (LC-preferential; *z*_LC_ = −3.32) to isorhamnetin (non-specific; *z*_LC_ = +0.74). Three of four contrasts moved the niche toward the autophagy–lysosome arm and one removed it, partially mitigating annotation-depth artefact as the sole explanation for the hydroxytyrosol–tyrosol contrast, though the tyrosol comparison remains limited by its small annotated target set. Pairwise topology analysis under the Cheng et al. [19] six-class scheme confirmed P1 Overlapping Exposure in 226 of 300 pairs (75.3%), with only 5 P2 Complementary Exposure pairs, all anchored on tyrosol (Fisher *p* = 2.17 × 10^−6^; Table S9), consistent with its minimal target set. The oleic acid + tyrosol pair (*s*_AB_ = +0.595) corresponds topologically to the MUFA and phenolic-alcohol composition of olive oil. The sub-module proximity results identify hydroxytyrosol as a computationally prioritised candidate nutraceutical; these are network-topological predictions that require experimental validation before biological conclusions can be drawn.

### 2.7. Molecular Docking Supports the Hydroxytyrosol–Tyrosol Structure–Activity Contrast at the Protein–Ligand Level

**Figure 8:**
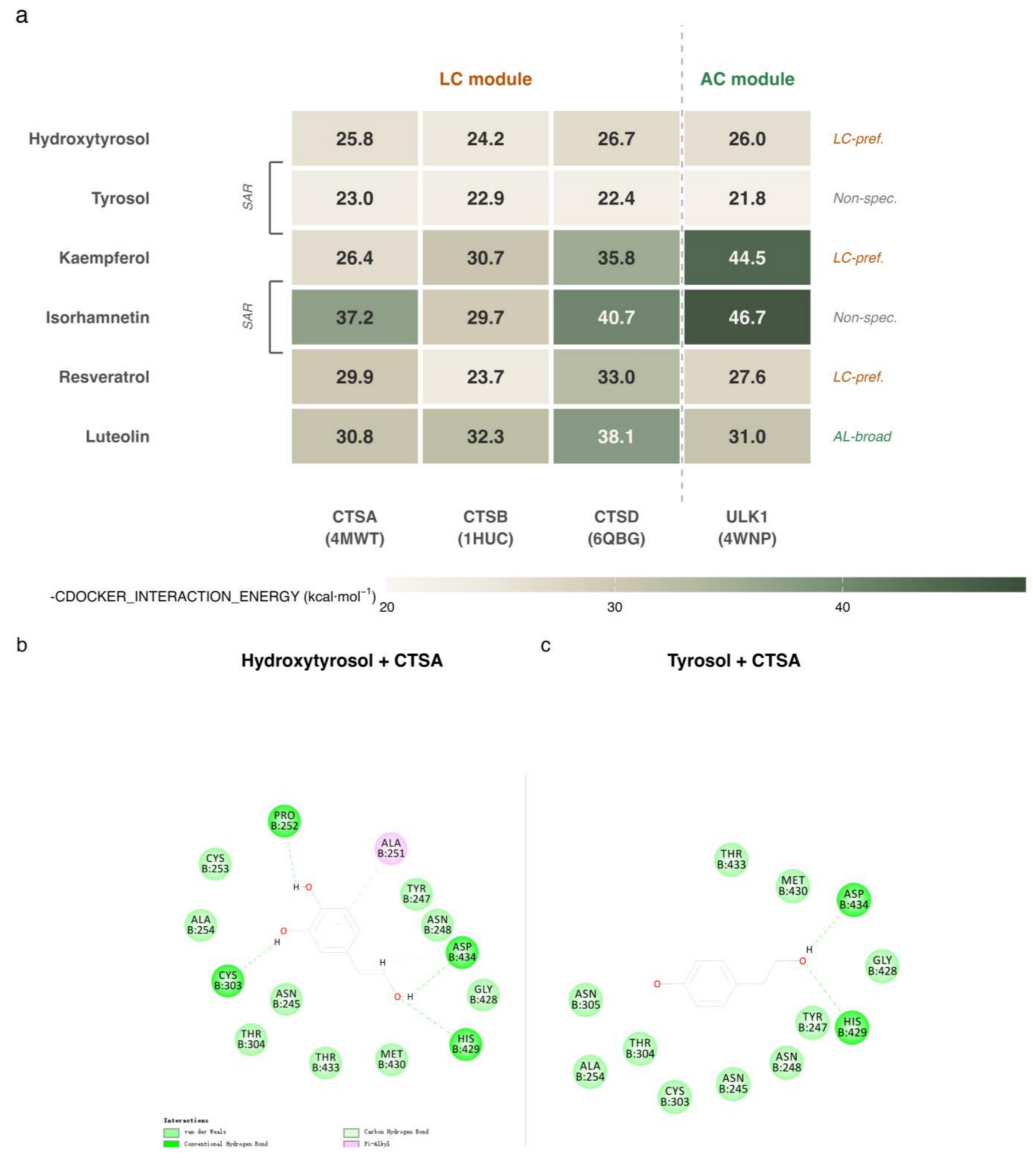
Molecular docking supports the hydroxytyrosol–tyrosol structure–activity contrast at the protein–ligand level. (**a**), Heatmap of −CDOCKER_INTERACTION_ENERGY (kcal mol^−1^) for six compounds docked against three lysosomal degradative-capacity (LC) module proteins (CTSA, PDB: 4MWT; CTSB, PDB: 1HUC; CTSD, PDB: 6QBG) and one autophagy core (AC) module protein (ULK1, PDB: 4WNP). Compounds are grouped by network-derived sub-module niche assignment (right labels). Brackets denote prespecified structure–activity pairs differing by a single chemical modification. Darker shading indicates higher interaction energy (stronger predicted binding). (**b**), Two-dimensional interaction diagram of hydroxytyrosol docked into the CTSA binding pocket (top-ranked pose by −CDOCKER_INTERACTION_ENERGY). Green dashed lines, conventional hydrogen bonds; light green circles, van der Waals contacts; pink circles, Pi-Alkyl interactions. Residue labels follow PDB chain and position numbering. (**c**), Corresponding diagram for tyrosol in the same CTSA binding pocket. Tyrosol forms two conventional hydrogen bonds (ASP B:434, HIS B:429) compared with four for hydroxytyrosol; the additional bonds in b originate from the ortho-hydroxyl group absent in tyrosol.

To test whether the network-derived sub-module assignments had a structural correlate in direct protein–ligand binding, we docked six compounds spanning three niche categories against four autophagy–lysosome proteins using the CDOCKER protocol (Figure 8a). Across all four targets, hydroxytyrosol consistently produced higher interaction energies than its structural congener tyrosol, which lacks the ortho-hydroxyl on the aromatic ring (mean difference +3.2 kcal mol^−1^; range +1.3 to +4.3 across CTSA, CTSB, CTSD and ULK1). Tyrosol returned the lowest energies of all six compounds (mean 22.5 kcal mol^−1^), in line with its non-specific sub-module assignment. Two-dimensional interaction analysis of the CTSA binding pocket illustrated the structural basis (Figure 8b,c): hydroxytyrosol formed four conventional hydrogen bonds (PRO B:252, CYS B:303, ASP B:434, HIS B:429), whereas tyrosol formed two (ASP B:434, HIS B:429). The two additional bonds in the hydroxytyrosol pose originated from the ortho-hydroxyl group, providing a molecular-level rationale for the single-hydroxyl-driven niche shift observed in the network analysis. This differential hydrogen-bond pattern is presented as a structural correlate of the network-derived niche difference rather than as evidence of functional modulation; confirmation by isothermal titration calorimetry (ITC) or surface plasmon resonance (SPR) of the recombinant CTSA–ligand interaction would be required to convert this structural observation into a measured binding affinity.

The second structure–activity pair did not follow the same pattern. Isorhamnetin produced higher interaction energies than kaempferol in three of four targets despite its non-specific network assignment, likely because the 3′-O-methyl group increases hydrophobic contact area within the binding pocket. This divergence between network proximity and docking affinity is expected: network proximity reflects the topological distribution of a compound’s annotated targets across the interactome, whereas docking estimates the physical complementarity of a single compound–protein pair. We further note that CDOCKER_INTERACTION_ENERGY is a relative ranking score rather than an absolute binding free energy, and the docking protocol employs a rigid receptor without explicit solvent; these results are therefore appropriate for intra-target comparative ranking but should not be interpreted as affinity predictions. Resveratrol and luteolin showed interaction profiles consistent with their LC-preferential and autophagy–lysosome-broad assignments, respectively, with luteolin achieving high scores across both LC and AC targets (Figure 8a). Together, the docking results provide structural-level support for the network-proximity-derived niche assignments as a hypothesis-generating framework; experimental validation of target engagement in cellular systems remains required before any functional conclusions can be drawn.

## 3. Discussion

Disabled macroautophagy and loss of proteostasis are recognised hallmarks of ageing [5], and the mTORC1-lysosome axis has emerged as a central node linking these processes in skin cell senescence models [9]. Much of the current mechanistic evidence, however, has been generated in experimentally induced senescence systems, and it remains unclear whether the autophagy–lysosome programme shifts during chronological ageing of primary human cells, and if so, at which molecular layer the shift is most apparent. Our paired transcriptome–proteome analysis of 82 GESTALT primary dermal fibroblast preparations spanning seven decades of healthy ageing [3,4] indicates that this shift is concentrated at the protein layer. The lysosomal degradative-capacity module, comprising cathepsins, V-ATPase subunits and lysosomal membrane proteins, carries the strongest and most consistent age association across all proteostasis modules tested, while the proteasome displays no directional age signal at the protein abundance level. Three proteins at the intersection of this module with the supervised multi-omics signature, TCIRG1, CTSA and ATP6V0D1, are each supported by four complementary analytical approaches drawing on the same GESTALT proteomic dataset and anchor the remodelling to a specific, experimentally tractable molecular neighbourhood. The protein-layer selectivity has broader implications for skin ageing research, because it indicates that transcript-level profiling alone, which currently dominates ageing biomarker discovery, systematically underestimates the extent to which the proteostasis landscape shifts with age in this cell type. This interpretation is further supported by the external GTEx transcriptomic comparison, in which neither anatomically matched nor photoaged skin tissue reproduced the module-resolved asymmetry at the mRNA level.

The age-dependent erosion of correspondence between transcript and protein levels is now documented in organisms from killifish to humans [10,11,13], and recent reviews have attributed this phenomenon to progressive translational constraints that reshape the proteome independently of transcriptional regulation [14,15]. The GESTALT fibroblast data sit within this framework. The global mRNA-protein correlation across 6995 paired genes was only 0.124, and per-donor coupling declined significantly with age. The mechanistic basis of this low global correspondence likely involves multiple post-transcriptional regulatory levels, including differential mRNA translation efficiency, heterogeneity in protein turnover rates, and microRNA-mediated translational repression; distinguishing among these mechanisms in the GESTALT cohort would require ribosome profiling or pulse-chase protein labelling approaches not available in the present dataset. By profiling a single mesenchymal cell type from inner-axillary biopsies at low passage, we sidestep the cell-type composition confounding that limits bulk-tissue studies [10] while preserving the cell-intrinsic ageing programme. The 13-tissue atlas of Ding et al. covered organ-level decoupling but did not resolve individual cell types [13]. A comparable mesenchymal-cell study showed protein synthesis downregulation in senescent mesenchymal stem cells [20]; our data extend this principle to chronologically aged primary dermal fibroblasts. The decoupling we observe is also not uniform across proteostasis subsystems. The autophagy-execution and lysosomal degradative-capacity gene sets lose mRNA-protein coupling significantly faster than the global gene set, whereas the proteasomal gene set does not lose coupling at all. This pathway-selective pattern is invisible in analyses that treat the transcriptome–proteome gap as a single aggregate measure. It is, however, in line with recent multi-layer profiling in the killifish brain, where aberrant translation elongation was shown to drive proteome remodelling independently of transcript changes, and where chronic proteasome impairment induced ageing-like lysosomal and mitochondrial signatures without recapitulating the global transcript–protein decoupling [12]. Our data extend that observation to human primary cells and show that the autophagy–lysosome and proteasome arms of the proteostasis network are differentially susceptible to protein-layer-dominant uncoupling, consistent with post-transcriptional regulation.

The accumulation of autophagy–lysosome proteins with age at the protein layer, against a flat proteasome, raises the question of whether this reflects an increase in autophagic flux or, alternatively, an expansion of autophagy–lysosome machinery without a corresponding gain in degradative throughput. Our cross-sectional design cannot resolve flux directly, but a mechanistic framework for the second interpretation is offered by the V-ATPase disassembly model recently described in yeast replicative ageing [21]. Based on published evidence from related cell systems, a speculative but testable mechanistic hypothesis (not established in the present dataset) is as follows: in that system, V-ATPase V1 and V0 subcomplexes progressively dissociate with age, with release of V1 subunit C from the lysosome-like vacuole into the cytosol; vacuolar pH rises, and caloric restriction prevents the disassembly. The mammalian V-ATPase is structurally conserved, with V1/V0 assembly regulated by mTOR signalling in fibroblasts [22,23]. Consistent with this model, lysosomal alkalinisation and impaired V-ATPase activity have been reported in senescent human fibroblasts [24]. TFEB nuclear translocation is transiently activated during acute cellular stress but becomes attenuated in established senescence when mTORC1 reactivates [25]. The transient-then-attenuated configuration is compatible with the protein-layer LC accumulation we observe in chronologically aged donors, although the stress-induced senescence model and our chronological cohort represent distinct categories of cellular ageing, and a direct equivalence cannot be assumed. Our WGCNA adds a proteome-level observation aligned with this picture. The V0-sector subunits TCIRG1, ATP6V0A2 and ATP6V0A4 co-segregate within the brown module (positively associated with age), while five V1-sector subunits (ATP6V1A, ATP6V1B2, ATP6V1E1, ATP6V1G1 and ATP6V1H) cluster in the separate midnightblue module. This sector-specific partitioning across two independently identified co-expression modules has not, to our knowledge, been reported in mammalian chronological ageing. Because the TMT proteomic quantification in the GESTALT dataset captures total cellular protein abundance rather than subcellular localisation, the co-expression divergence between V0 and V1 subunits cannot be directly equated with physical V1 release from lysosomes as reported in yeast [21]. Autophagic flux cannot be directly assessed from static proteomic snapshots; the cross-sectional TMT data are consistent with an accumulation of autophagy–lysosome machinery but do not discriminate between increased flux, impaired flux with compensatory biogenesis, and sequestration of non-functional protein complexes. The partitioning is, however, consistent with a broader framework in which V0 and V1 follow distinct regulatory trajectories during ageing [8,24], and it identifies specific subunits whose subcellular redistribution can be directly interrogated in primary fibroblast models.

Network medicine approaches to drug repurposing have shown that disease-associated genes form connected interactome modules and that compound proximity to such modules predicts therapeutic relevance better than direct target overlap [16,17]. Applications of this principle to ageing have so far tended to operate at the pan-hallmark, tissue-agnostic scale, which captures longevity-associated gene-compound proximity at the level of whole biological programmes but does not resolve intra-module heterogeneity at the cell-type level. Our 244-protein target module is anchored on a data-driven, cell-type-specific multi-omics signal rather than on a curated longevity gene list, and so reflects the ageing programme of dermal fibroblasts specifically. The sub-module decomposition into lysosomal degradative-capacity (M_LC), autophagy core (M_AC) and chromatin/splicing (M_chromatin) arms adds intra-module resolution and is consistent with compound-level niche assignment. The observation that 72% of top 25 nutraceutical leads are topologically anchored on the autophagy–lysosome arm mirrors the proteomic result showing LC as the dominant age-remodelled sub-system, and suggests that the network prioritisation is capturing biologically relevant target neighbourhoods rather than non-specific whole-module proximity.

Four prespecified intra-family contrasts tested whether niche assignment is sensitive to defined chemical modifications. Introducing the ortho-hydroxyl that distinguishes hydroxytyrosol from tyrosol shifted the compound from non-specific to LC-preferential. Introducing C2 and C3 unsaturation (naringenin to luteolin) shifted the niche from non-specific to autophagy–lysosome-broad. Extending the fatty-acid chain by two methylene units (palmitoleic acid to oleic acid) moved the compound into the LC-preferential niche. Conversely, 3′-O-methylation (kaempferol to isorhamnetin) abolished the LC-preferential signal. Three of the four modifications moved the niche toward the autophagy–lysosome direction and one removed it, supporting the interpretation that sub-module assignment may reflect structure-dependent biology in this network context. The kaempferol/isorhamnetin pair, with comparable annotated target-set sizes (146 versus 125), is the most informative contrast because it is minimally confounded by annotation depth.

Among the autophagy–lysosome-directed leads, hydroxytyrosol (HT) merits closer attention for several converging reasons. Based on published evidence from related cell systems, a plausible mechanistic hypothesis, not yet established in the present dataset, is that HT modulates a signalling network in which SIRT1, AMPK and Akt/mTORC1 converge on TFEB-mediated lysosomal biogenesis. In vascular adventitial fibroblasts, HT upregulates SIRT1 at both the mRNA and protein levels, increases LC3-II conversion and Beclin1 expression, and suppresses Akt and mTOR phosphorylation in a SIRT1-dependent manner; SIRT1 siRNA knockdown abrogated the autophagy-promoting effect when HT was applied at 50 μM together with TNF-α stimulation, placing SIRT1 upstream of the autophagic readout [26]. HT-induced AMPK activation and autophagy induction have been compiled across cancer, metabolic syndrome, osteoporosis, immune-mediated and neurodegenerative models [27]. In pre-senescent human dermal (NHDF) and lung (MRC5) fibroblasts, chronic treatment with 1 μM HT for 4 to 6 weeks reduces β-galactosidase-positive cell number, p16 expression and SASP markers including IL-6, metalloprotease secretion and COX-2 [28], indicating that the network operates at exposures compatible with realistic dietary intake. Our sub-module proximity analysis places HT’s annotated target set within the V-ATPase, cathepsin and LAMP neighbourhood of M_LC (z_LC = −3.27), a topology consistent with the path from upstream SIRT1, AMPK and mTOR signalling to TFEB-driven CLEAR-network transcriptional output [6]. No published wet-lab study, however, directly tests HT-induced TFEB nuclear translocation, HT modulation of lysosomal pH or HT-induced changes in cathepsin activity in primary mammalian cells. These are testable predictions that the integrated network medicine and docking pipeline generates rather than confirms, and that the HT pharmacology summarised above renders mechanistically plausible. This computational prioritisation is consistent with a role for HT as a downstream LC engager in dermal fibroblasts, not only an antioxidant or anti-inflammatory, though direct experimental evidence in chronologically aged primary fibroblasts is lacking. HT already carries dietary regulatory endorsement under Commission Regulation (EU) No 432/2012. HT is a principal polyphenol of extra-virgin olive oil, with an estimated daily intake of between 0.15 and 30 mg in the context of Mediterranean dietary patterns [27]. The pairwise topology analysis further identifies oleic acid and tyrosol as a Complementary Exposure pair, providing a network-level proxy for the combined MUFA and phenolic-alcohol composition of extra-virgin olive oil converging on the autophagy–lysosome arm from complementary sub-module angles.

The contrast between hydroxytyrosol and tyrosol also has a structural correlate at the level of direct interaction with target proteins. We used the CDOCKER algorithm, which combines high-temperature simulated annealing with rigid-receptor all-atom minimisation [29]. Across four autophagy–lysosome targets (CTSA, CTSB, CTSD and ULK1), hydroxytyrosol produced higher CDOCKER interaction energies than tyrosol, with a mean differential of +3.2 kcal mol^−1^. Two-dimensional contact analysis of the CTSA binding pocket identified the chemical basis. Hydroxytyrosol forms four conventional hydrogen bonds (PRO B:252, CYS B:303, ASP B:434 and HIS B:429), tyrosol forms two (ASP B:434 and HIS B:429), and the two additional contacts arise from the ortho-hydroxyl group that is the single chemical feature distinguishing the two compounds. The interpretation has limits. CDOCKER INTERACTION_ENERGY is a relative ranking score, not an absolute binding free energy. The scoring function carries known biases including a preference for larger ligands, the absence of explicit water modelling, and limited treatment of induced fit beyond local side-chain rearrangement. The differential of four versus two hydrogen bonds should therefore be read as a structural fingerprint of the catechol contact mode at CTSA rather than as a ΔΔG estimate. Confirmation by isothermal titration calorimetry (ITC) or surface plasmon resonance (SPR) of the recombinant CTSA–ligand interaction, or by a free-energy-perturbation framework, would convert this fingerprint into a measured affinity and remains an experimental step. The network and docking results converge on the same chemical feature. The single ortho-hydroxyl that re-routes hydroxytyrosol into the LC sub-module also extends the hydrogen-bond network at the lysosomal protease binding site, and CTSA is the most tractable single-target entry point for biochemical follow-up.

The 13 dual-track convergence candidates do not overlap Gene Ontology autophagy terms in either omics block (Fisher’s exact test, protein *p* = 0.60, transcript *p* = 1.00), yet GSEA of the full proteome ranking identified macroautophagy and vacuole organisation as the top enriched terms in the age-upregulated direction.This apparent discrepancy reflects a well-recognised difference between enrichment frameworks [30]. Dual-track candidates were selected for maximal individual discriminative power, criteria that favour large-effect genes over pathway-wide patterns, whereas GSEA detects distributed, moderate-amplitude signals across the full ranked list. Autophagy–lysosome remodelling is instead carried by coordinated moderate shifts distributed across hundreds of module members, consistent with a pathway-level functional decline rather than the collapse of individual sentinel genes [5]. The two analytical layers are therefore complementary rather than contradictory. The autophagy–lysosome module provides the systems-level biological context, while the dual-track candidates, led by NBEAL1, FKBP11, TMOD2 and LSM14B at the top of the composite evidence ranking, provide individual molecular handles whose functional roles in dermal ageing have not been characterised and merit independent investigation.

Several limitations frame the interpretation of this work. The study is a computational reanalysis of existing GESTALT paired multi-omics data [3,4], and all conclusions are correlative. The cohort comprises 82 donors of a single cell type from one anatomical site (inner axillary skin), and the fibroblasts were analysed after short-term culture at low passage. Although this design preserves cell-intrinsic ageing programmes while minimising in vitro artefacts, it cannot capture microenvironment-dependent effects that operate in intact tissue. Dermal fibroblasts comprise functionally distinct subpopulations that erode with age [31]; whether the autophagy–lysosome remodelling we describe is subtype-specific remains an open question for future single-cell or spatial proteomic studies. TMT-based quantification at the MS2 level is subject to ratio compression [32], which biases reported effect sizes toward the null and reduces the sensitivity of single-protein tests relative to module-level analyses; the proteomic differentials we report should be read as conservative lower bounds rather than calibrated effect magnitudes. The WGCNA modules showed high within-cohort preservation statistics, but the brown and turquoise modules have not been validated in an independent fibroblast proteome dataset, and the midnightblue module weakened below the FDR threshold upon removal of a single outlier donor. The supporting evidence for TFEB and V-ATPase derives primarily from senescence models [25,33] rather than chronologically aged primary cells; direct alignment with the GESTALT cohort still requires experimental confirmation. Furthermore, the network medicine and docking predictions cannot guarantee in vivo efficacy; validation first in primary fibroblasts derived from aged donors under physiologically relevant conditions and ultimately in appropriate in vivo models remains essential before translational conclusions can be drawn.Future experimental priorities include lysosomal pH measurement, cathepsin B/D activity assays, LC3-II/p62 turnover, TFEB nuclear localisation, and hydroxytyrosol treatment of aged primary dermal fibroblasts at senomorphic concentrations, as previously described [28].

The nutraceutical prioritisation inherits annotation biases from the underlying target databases. Compounds with larger experimentally characterised target sets are more likely to reach significant network proximity, and the curated longevity evidence used in the composite prioritisation score draws predominantly on invertebrate lifespan assays [34]. Niche assignments for compounds with fewer than ten annotated targets, including tyrosol and butein, should be interpreted accordingly; the kaempferol/isorhamnetin pair, with comparable target-set sizes, provides the most annotation-balanced structure-activity comparison.

Even with these caveats, the convergence of four complementary analytical approaches drawing on the same GESTALT proteomic dataset on the same molecular neighbourhood, combined with an external transcriptomic comparison in a GTEx skin cohort, supplies a degree of triangulation that is uncommon in single-cohort computational studies. A computational hypothesis follows from this work: that hydroxytyrosol may engage the lysosomal degradative-capacity sub-module of the dermal fibroblast ageing programme by acting on the SIRT1, AMPK and mTOR network that controls TFEB-driven transcription of V-ATPase subunits and cathepsins, with CTSA as the most tractable single-target entry point. More broadly, the integration of cell-type-resolved ageing proteomics with network medicine-guided nutraceutical prioritisation reported here could be extended to other skin cell populations, to fibroblasts from photoaged or inflammatory contexts, and to emerging spatial proteomic platforms that resolve both molecular identity and tissue localisation in the same section. Such extensions would test whether the autophagy–lysosome remodelling signature identified here generalises beyond the inner-axillary fibroblast system and whether the computationally prioritised candidate nutraceuticals nominated here retain their sub-module specificity across ageing contexts.

## 4. Materials and Methods

### 4.1. Study Cohort and Data Sources

This study reanalysed paired transcriptomic and proteomic data from the GESTALT cohort (*n* = 82 clinically healthy participants, aged 22–89 years), in which primary fibroblast cultures were derived from non-sun-exposed inner axillary skin biopsies [3,4]. RNA-seq counts (GEO accession GSE226189) and TMT proteomic abundances (MassIVE accession MSV000088401) were generated from the same donor-derived cell preparations. For supervised integration, participants were stratified into three age tertiles: Young (22–42 years, *n* = 30), Middle (43–65 years, *n* = 25) and Old (66–89 years, *n* = 27); sex balance across groups was confirmed by chi-square test.

RNA-seq counts were variance-stabilised with the DESeq2 variance-stabilising transformation (VST) (blind = FALSE) [35] and filtered for protein-coding genes (EnsDb.Hsapiens.v86 [36]; ≥10 counts in at least as many samples as the smallest age group), yielding 15,666 genes. In the TMT matrix, proteins missing in >50% of samples were removed and remaining values imputed by *k*-nearest neighbours (*k* = 10) [37]. Both matrices were sex-corrected with limma::removeBatchEffect [38], retaining age group as a protected factor. Age-group separation was verified by PERMANOVA (vegan::adonis2, Euclidean distance, 999 permutations) [39]. Additional preprocessing parameters are provided in Supplementary Methods. All statistical analyses were performed using R version 4.5.2 (R Foundation for Statistical Computing, Vienna, Austria).

### 4.2. Transcriptome–Proteome Decoupling and Quadrant Enrichment

Decoupling was quantified across 6995 matched gene–protein pairs mapped through the study-level Ensembl–UniProt table. For each pair, standardised age-regression coefficients (*β*_mRNA_ and *β*_protein_) were obtained from separate ordinary least-squares models with chronological age as the predictor. The decoupling score was the *Z*-standardised residual from a global regression of *β*_protein_ on *β*_mRNA_, with BH correction [40]; |*Z*| > 2 at adjusted significance defined candidate targets. A prespecified DEGRADED-mode criterion applied when the Pearson correlation between the decoupling score and *β*_protein_ exceeded 0.95; candidates were then reinterpreted as protein-layer ageing outliers rather than strict decoupling targets (sensitivity thresholds in Supplementary Methods). Under DEGRADED mode, the residual-based decoupling score reduces to a protein age-regression coefficient; the analysis therefore primarily identifies proteins with strong age-associated abundance changes rather than genes exhibiting genuine mRNA–protein decoupling.

All matched genes were assigned to four quadrants by the signs of *β*_mRNA_ and *β*_protein_ (Q1–Q4). Over-representation analysis (ORA) of the two discordant quadrants (Q2 and Q4) was performed with fgsea::fora [41] against the full matched-gene background [42,43], using GO Biological Process and KEGG gene sets from the msigdbr R package (version 26.1.0; MSigDB release 2026.1.Hs) [44] (size 15–500; BH FDR < 0.05; GO redundancy reduced by Wang semantic similarity at threshold 0.70 [45]).

### 4.3. Per-Donor Coupling Index and Pathway-Resolved Trajectories

For each donor, the mRNA–protein coupling index was defined as the Spearman correlation across all paired genes. Donors were divided at the cohort median age (52 years) into lower-age (*n* = 42) and higher-age (*n* = 40) groups and compared by two-sided Mann–Whitney *U* test with rank-biserial effect size [46]. Coupling was also regressed on continuous age to obtain a standardised *β*_Age_ with 95% confidence interval (CI). A sliding-window analysis (5-year width, 1-year step) characterised the age trajectory of coupling across the lifespan.

This framework was then applied to five prespecified gene subsets spanning autophagy execution, autophagy flux, the proteasome and the ribosome (negative control), defined from the published Autophagy Gene Toolbox gene lists (version of record published 2 November 2021) [47] and KEGG pathway annotations included in MSigDB release 2026.1.Hs and retrieved with the msigdbr R package (version 26.1.0) [48] (subset compositions listed in Supplementary Table S1). Whether autophagy-execution coupling decline exceeded the global or proteasomal baseline was tested by stratified bootstrap resampling within age groups (*B* = 1000), with one-sided *p* values BH-adjusted across 12 planned comparisons [40].

### 4.4. Supervised Integration and Candidate Selection

DIABLO, a supervised multi-block sparse integration method [49,50], was applied to the transcriptomic and proteomic matrices with the three age tertiles as outcome. The design matrix was selected by comparing null and low-covariance configurations under repeated stratified 5-fold cross-validation (50 repeats), evaluated by balanced error rate (BER) under the centroid-distance classifier. At most, two components were retained. Block-specific feature counts (keepX) were tuned with tune.block.splsda under the same cross-validation scheme (tuning grids and decision rules in Supplementary Methods). Selection stability per feature was the proportion of cross-validation folds in which it appeared. Feature selection was performed within each cross-validation fold, ensuring that held-out test data were not accessed during feature selection and preventing information leakage. Age-label permutation testing (*n* = 100 permutations; 5-fold cross-validation per permutation) confirmed above-chance classification performance (Figure S3e; Supplementary Methods). Single-omics age associations were fitted independently, with transcriptomic effects estimated by the limma empirical-Bayes framework [38,51] and proteomic effects by ordinary least-squares regression, both BH-corrected.

Candidates were assembled through two complementary tracks. Track 1 comprised all features selected by DIABLO across retained components. Track 2 drew on the decoupling analysis through a tiered thresholding strategy (Tiers A–C; criteria in Supplementary Methods), targeting a combined candidate set of 80–120 features; under DEGRADED mode, Track 2 captured protein-layer ageing outliers. Features appearing in both tracks were dual-track convergence candidates. To integrate evidence from the two selection strategies, candidates were ranked by a composite evidence score (CES) following rank aggregation principles [52]:

$$CES=0.5\times {Stability}_{norm}+0.3\times {Loading}_{norm}+0.2\times {Decoupling}_{norm}$$

The heaviest weight was assigned to cross-validation selection stability, the most direct measure of reproducible age-group discrimination in DIABLO; loading magnitude and decoupling evidence received progressively lower weights as complementary indicators. Each term was min–max normalised to [0, 1]; the decoupling term was set to zero under DEGRADED mode. Robustness of the CES ranking to the weighting scheme was confirmed under two alternative configurations (Supplementary Methods).

### 4.5. Pathway Enrichment and Module-Targeted Age Regression

Gene set enrichment analysis (GSEA) [30] was performed with fgsea on three feature rankings: Pearson correlation with DIABLO first-component scores (per omics block), continuous-age mRNA association and proteomic *t* statistics from sex-adjusted age regression [51]. Gene sets from GO Biological Process, Hallmark and Reactome collections [44,53] were restricted to size 15–500 (≥ 10,000 permutations; BH FDR < 0.05). Over-representation of dual-track candidates within the autophagy reference set (GO:0006914 ∪ GO:0016236 [54]) was assessed by one-sided Fisher’s exact test.

Three proteostasis modules were prespecified: the autophagy core (AC) module (22 proteins from the Gene Toolbox autophagy core and docking/fusion categories [47]), the lysosomal degradative-capacity (LC) module (15 proteins spanning cathepsins, LAMP1/2 and V-ATPase subunits [22]; 13 quantified in the GESTALT proteome) and the proteasomal (PS) module (KEGG hsa03050 [48]). Full member lists are provided in Table S10. For each module member, sex-corrected protein abundance was regressed on continuous age, and module-level distributions of standardised age coefficients were compared by Kruskal–Wallis test followed by Dunn post hoc comparisons [55] with Benjamini–Hochberg (BH) correction.

Layer asymmetry was tested by intersecting each module with the matched transcript–protein set. For each gene, age-association significance (FDR < 0.05) was recorded separately at the protein and transcript layers. The exact conditional McNemar test [56] assessed whether the count of genes significant only in the proteome (*n*_10_) differed from those significant only in the transcriptome (*n*_01_); the matched-pairs odds ratio *n*_10_/*n*_01_ exceeding 1 indicated a protein-layer-dominant signal. Because the exact conditional test is conservative at small discordant counts [57], a prespecified sensitivity analysis relaxed the protein threshold to FDR < 0.10.

### 4.6. Weighted Protein Co-Expression Network Analysis

A signed hybrid weighted co-expression network was constructed from the sex-corrected proteomic matrix using the WGCNA R package (version 1.74) [58] with biweight midcorrelation as the similarity measure [59]. The soft-thresholding power β was the smallest value at which the scale-free topology fit index exceeded 0.80 and entered a stable plateau [60]. Modules were detected by hybrid dynamic tree cutting on the topological overlap dissimilarity matrix (minModuleSize = 30, deepSplit = 2, mergeCutHeight = 0.25); proteins assigned to the grey module were excluded. Outlier detection, hub-recovery sensitivity analysis and module preservation procedures are detailed in Supplementary Methods. Sensitivity analyses evaluating the effect of soft-thresholding power (β ± 1 unit), minimum module size (20 and 40 proteins), correlation metric and missing-value imputation strategy on module composition are also described in Supplementary Methods. The absence of an independent fibroblast proteome validation cohort is acknowledged as a limitation.

For each non-grey module, the Spearman correlation between the module eigengene (ME) and continuous age was BH-adjusted across modules [40]; FDR < 0.05 defined age-associated modules. Hub proteins within these modules satisfied module membership (MM) ≥ 0.80 and gene significance for age (GS_age_) ≥ 0.30 [61]. Functional enrichment per age-associated module used fgsea::fora [41] against all quantified proteins, with GO Biological Process and KEGG gene sets from msigdbr [44,48] (size 15–500; BH FDR < 0.05; GO redundancy reduced by Wang semantic similarity at 0.70 [45]). Convergence between unsupervised WGCNA modules and supervised DIABLO candidates was tested by one-sided Fisher’s exact test.

### 4.7. GTEx v8 Transcriptomic Validation

GTEx v8 [62] served as an external tissue-level transcriptomic reference to test whether the protein-layer AC and LC remodelling observed in GESTALT fibroblasts was mirrored at the transcript layer in whole skin. Two sites were analysed: Skin—Not Sun Exposed (Suprapubic, *n* = 639), anatomically closest to the GESTALT biopsy site, and Skin—Sun Exposed (Lower Leg, *n* = 768). Because GTEx whole-skin samples contain multiple cell types, use age-bin encoding rather than continuous age, and are subject to post-mortem variables, this comparison provides tissue-level context rather than direct validation of the GESTALT fibroblast findings. Donor age, released in decade bins, was encoded by bin midpoints (25–75 years). Genes with counts per million (CPM) ≥ 1 in ≥ 40% of samples were retained [63], normalised by the trimmed mean of M-values (TMM) method [64] as implemented in edgeR [65] and voom-transformed [38,66]. The design matrix included three peri-mortem covariates (RNA integrity, Hardy death scale and ischaemic time) [67] and surrogate variables estimated by the sva permutation procedure [68]. Age association per gene was modelled by limma empirical Bayes with BH adjustment within each tissue. A prespecified sensitivity analysis on the fast-death subset (Hardy 1 or 2) assessed robustness to peri-mortem confounding (Supplementary Methods).

The three GESTALT proteostasis modules (AC, LC and PS) were mapped to GTEx at the HGNC symbol level; 72 of 73 members were recovered in both tissues. Module-level transcript age effects were compared by Kruskal–Wallis test with Dunn post hoc comparisons [55] (BH-corrected), mirroring the proteomic-layer framework. Gene set variation analysis (GSVA) [69] provided complementary sample-level enrichment scores correlated with age. The interpretive boundary was fixed before analysis: weak or absent AC/LC > PS asymmetry at the transcript layer would be reported as consistent with protein-layer-dominant remodelling, rather than as a failed replication.

### 4.8. Network Medicine Compound Prioritisation

All compound rankings were treated as in silico priors for downstream experimental testing, not as evidence of therapeutic efficacy [17,70]. Compound–target databases are incomplete and biased toward extensively studied molecules; network proximity scores reflect current annotation coverage and represent computational prioritisation signals rather than evidence of biological activity. The ageing target module (244 proteins) comprised a Core module (intersection of DIABLO candidates and WGCNA age-significant robust hubs) and an Extended module (single-framework support at prespecified secondary confidence; assembly criteria in Supplementary Methods). Network agglomeration was verified by comparing the observed largest connected component (LCC) size against 10,000 degree-matched random gene sets [16]; empirical *z* > 1.96 indicated significant clustering.

The background interactome was the human STRING v12.0 network (combined score ≥ 0.700; LCC: 15,882 nodes, 473,424 edges) [71]. A candidate compound library (*n* = 341) was compiled from geroprotector databases [34,72] and the recent literature, with experimentally supported targets drawn from ChEMBL (activity ≤ 10 μM) [73], STITCH v5 (combined score ≥ 0.700) [74] and SwissTargetPrediction (top 15 predicted targets) [75]; compounds with fewer than 3 or more than 200 mapped targets were excluded [17]. Subsequent analyses were restricted to the nutraceutical subset (*n* = 58, 17.0%; library assembly and class-annotation procedures in Supplementary Methods).

Network proximity between each compound target set and the ageing module was quantified by the closest-distance metric of Guney et al. [17], with significance assessed against 1000 degree-matched random reference sets. Following the composite reranking logic applied in network-based drug-repurposing pipelines [76], compounds were ranked by a composite prioritisation score (CPS):

$$CPS=0.5\times R_{proximity}+0.3\times R_{ORA}+0.2\times R_{evidence}$$

The heaviest weight was assigned to network proximity, the central predictor of drug–module perturbation in validated network medicine screens [16,17]; direct target overlap and curated longevity evidence served as complementary terms. Each component was rank-scaled to [0, 1]; robustness was confirmed across three alternative weighting schemes (Supplementary Methods).

To resolve sub-module-specific signal within the 244-protein module, three sub-modules were carried forward without redefinition: M_LC_ (15 lysosomal degradative-capacity proteins [22]), M_AC_ (29 autophagy core proteins [47]) and M_chromatin_ (21 chromatin/splicing proteins anchored on the four Core-module hubs and their first-shell interactome partners). Member lists are in Supplementary Table S11. For each top 25 CPS nutraceutical, closest-distance proximity to each sub-module was computed against 500 degree-matched random sets [16]; *z* ≤ −2 defined significant proximity, and each compound was assigned to a functional niche based on the pattern of sub-module *z*-scores (LC-preferential, AC-preferential, autophagy–lysosome-broad, chromatin-preferential, broad-coverage or non-specific; definitions in Supplementary Methods). A prespecified intra-family comparison between hydroxytyrosol and tyrosol [77], differing by a single hydroxyl group, tested whether close chemical congeners occupied distinct niches. Pairwise network-based separation *s*_AB_ [16,19] among all top 25 pairs was computed to assess combination potential (topology-class rules in Supplementary Methods).

### 4.9. Molecular Docking

To test whether network-derived sub-module assignments had a structural correlate at the protein–ligand level, six compounds spanning three niche categories were docked against four autophagy–lysosome hub proteins using the CDOCKER algorithm [29] implemented in BIOVIA Discovery Studio 2019 (Dassault Systèmes BIOVIA, San Diego, CA, USA). Three LC module targets were selected from the upstream multi-omics convergence: cathepsin A (CTSA; PDB: 4MWT), cathepsin B (CTSB; PDB: 1HUC) and cathepsin D (CTSD; PDB: 6QBG). ULK1 (PDB: 4WNP) represented the AC module as the sole member with a co-crystallised small-molecule binding pocket. Structures were prepared with CHARMm force-field typing [78]; binding sites were defined from co-crystallised ligand coordinates (CTSD, ULK1; 13 Å radius) or by receptor cavity detection (CTSA, CTSB).

Three LC-preferential compounds (hydroxytyrosol, kaempferol and resveratrol), one autophagy–lysosome-broad compound (luteolin) and two non-specific controls (tyrosol and isorhamnetin) were docked, forming two prespecified structure–activity pairs differing by a single functional group (ortho-hydroxyl and 3′-O-methyl, respectively). CDOCKER generated ten random conformations per protein–ligand pair through high-temperature dynamics (700 K, 2000 heating steps) followed by simulated-annealing refinement (5000 cooling steps); the top-ranked pose by −CDOCKER_INTERACTION_ENERGY (kcal/mol) was retained. Two-dimensional interaction diagrams were generated in Discovery Studio. CDOCKER employs a rigid receptor without explicit solvent modelling and generates interaction energies rather than binding free energies; results are appropriate for relative intra-target comparative ranking but should not be interpreted as absolute affinity predictions or as evidence of functional engagement in cells.

## 5. Conclusions

Analysis of 82 GESTALT primary dermal fibroblasts across seven decades of healthy ageing reveals that age-associated proteostasis remodelling is concentrated at the protein layer, with the lysosomal degradative-capacity module carrying the strongest and most consistent age signal across all proteostasis arms examined. The proteasome shows no corresponding directional shift. Three proteins, TCIRG1, CTSA and ATP6V0D1, converge across complementary supervised, unsupervised and module-targeted analytical frameworks and provide specific molecular entry points into this remodelling. The absence of a matching transcript-level asymmetry in external GTEx skin data is consistent with protein-layer-dominant remodelling of the autophagy–lysosome axis.

Network medicine prioritisation of the 244-protein ageing proteostasis module nominates hydroxytyrosol as a computationally prioritised candidate nutraceutical with preferential topological proximity to the lysosomal degradative-capacity sub-module, a niche assignment corroborated at the structural level by molecular docking. As this work is entirely computational, all predictions require experimental confirmation in aged primary fibroblasts before functional conclusions can be drawn. The cell-type-resolved multi-omics and network medicine framework described here is directly transferable to other skin cell populations and ageing contexts.

## Data Availability

All data analysed in this study are publicly available. Paired RNA-seq counts are deposited in the Gene Expression Omnibus under accession GSE226189. Tandem mass tag proteomic abundances are deposited in the MassIVE repository under accession MSV000088401. GTEx v8 transcriptomic data were obtained from the GTEx Portal (https://www.gtexportal.org/). Analysis code is available from the corresponding author upon reasonable request.

## Supplementary Materials

The following supporting information can be downloaded at: https://www.mdpi.com/article/10.3390/ijms27156808/s1. References [3,5,16,17,19,22,32,34,39,40,41,47,48,49,58,60,68,69,72,73,74,75,76,77,78] are cited in the Supplementary Materials.
