# Supplementary material for: Proteome-Level Autophagy–Lysosome Remodelling Marks Ageing in Human Dermal Fibroblasts and Nominates Hydroxytyrosol as a Candidate Nutraceutical
Source: Int J Mol Sci. 2026 Jul 29;27(15):6808. doi: 10.3390/ijms27156808 (PMC13467536; doi:10.3390/ijms27156808)
Supplement: Supplementary file 1 [file ijms-27-06808-s001.zip › Supplementary Figure S1. ORA_Q2Q4_GOBP.pdf]

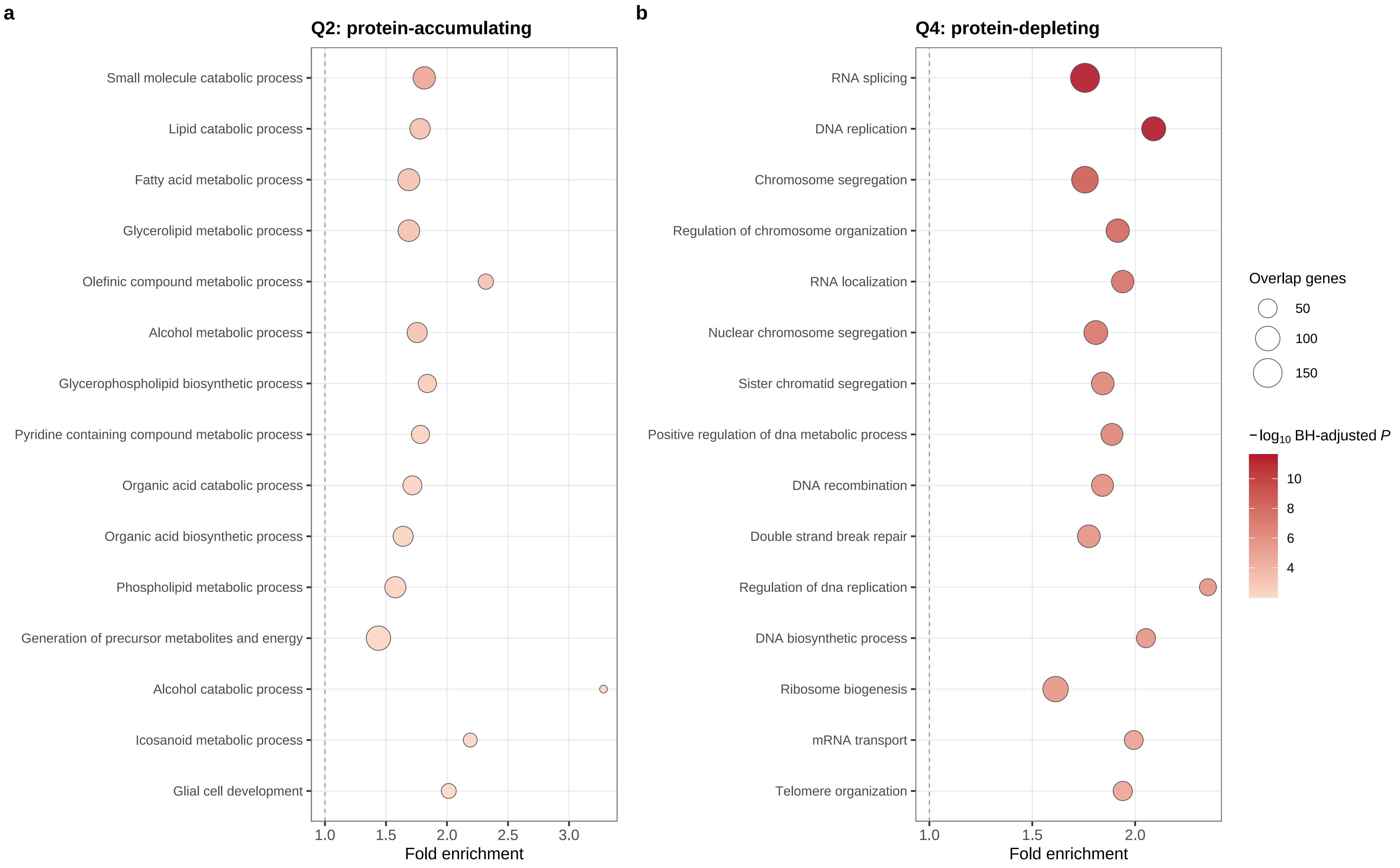
a
Q2: protein-accumulating
Small molecule catabolic process
Lipid catabolic process
Fatty acid metabolic process
Glycerolipid metabolic process
Olefinic compound metabolic process
Alcohol metabolic process
Glycerophospholipid biosynthetic process
Pyridine containing compound metabolic process
Organic acid catabolic process
Organic acid biosynthetic process
Phospholipid metabolic process
Generation of precursor metabolites and energy
Alcohol catabolic process
Icosanoid metabolic process
Glial cell development
1.0
1.5
2.0
2.5
3.0
Fold enrichment
b
Q4: protein-depleting
RNA splicing
DNA replication
Chromosome segregation
Regulation of chromosome organization
RNA localization
Nuclear chromosome segregation
Sister chromatid segregation
Positive regulation of dna metabolic process
DNA recombination
Double strand break repair
Regulation of dna replication
DNA biosynthetic process
Ribosome biogenesis
mRNA transport
Telomere organization
1.0
1.5
2.0
Fold enrichment
Overlap genes
50
100
150
$-\log_{10}$ BH-adjusted P
10
8
6
4
