# Supplementary material for: Proteome-Level Autophagy–Lysosome Remodelling Marks Ageing in Human Dermal Fibroblasts and Nominates Hydroxytyrosol as a Candidate Nutraceutical
Source: Int J Mol Sci. 2026 Jul 29;27(15):6808. doi: 10.3390/ijms27156808 (PMC13467536; doi:10.3390/ijms27156808)
Supplement: Supplementary file 1 [file ijms-27-06808-s001.zip › Supplementary Figure S2a. PCA_mRNA_age_groups.pdf]

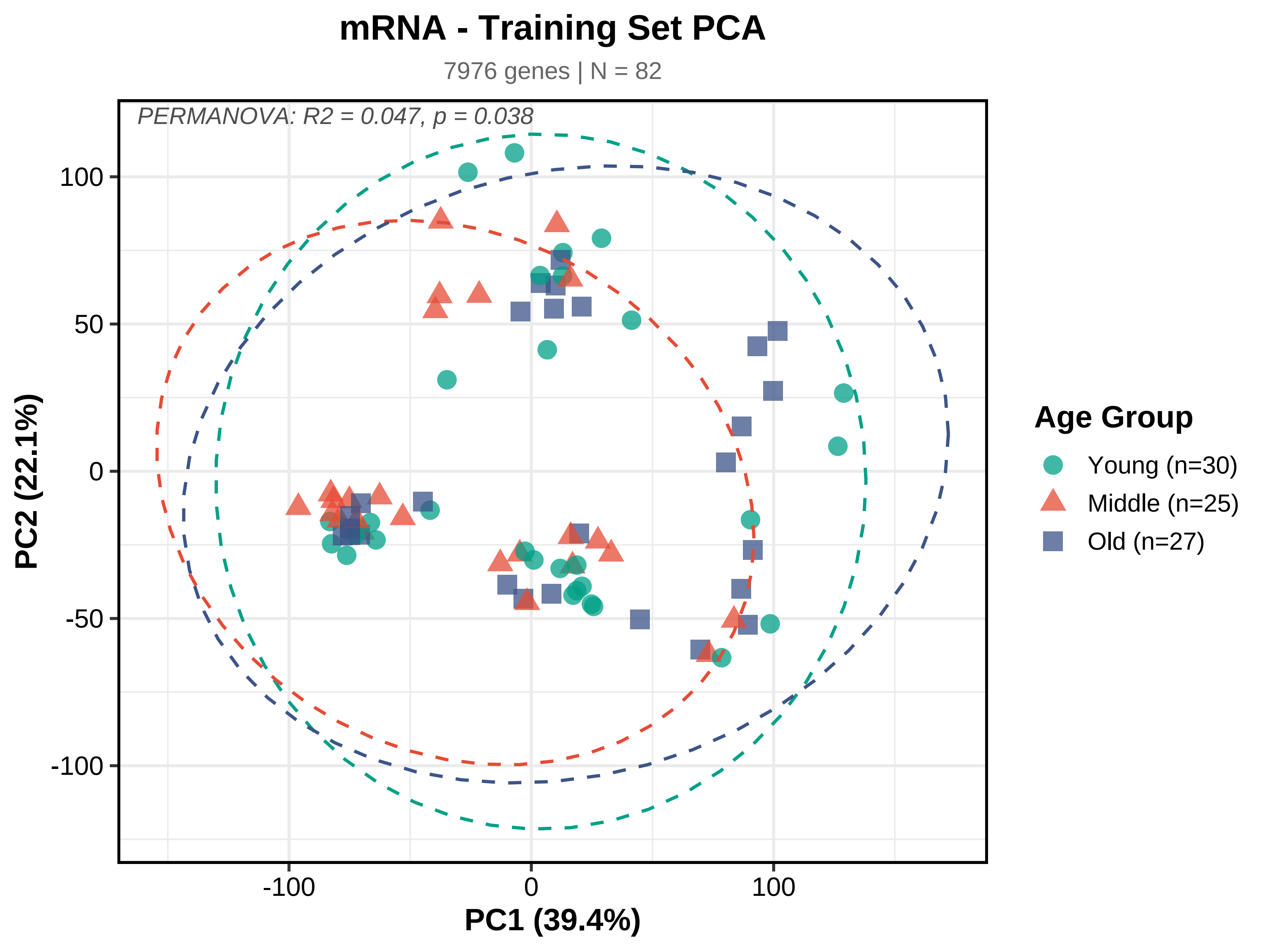
mRNA - Training Set PCA
7976 genes | N = 82
PERMANOVA: R2 = 0.047, p = 0.038
100
50
0
-50
-100
PC2 (22.1%)
-100
0
100
PC1 (39.4%)
Age Group
Young (n=30)
Middle (n=25)
Old (n=27)
