# Supplementary material for: Proteome-Level Autophagy–Lysosome Remodelling Marks Ageing in Human Dermal Fibroblasts and Nominates Hydroxytyrosol as a Candidate Nutraceutical
Source: Int J Mol Sci. 2026 Jul 29;27(15):6808. doi: 10.3390/ijms27156808 (PMC13467536; doi:10.3390/ijms27156808)
Supplement: Supplementary file 1 [file ijms-27-06808-s001.zip › Supplementary Figure S2b. PCA_Protein_age_groups.pdf]

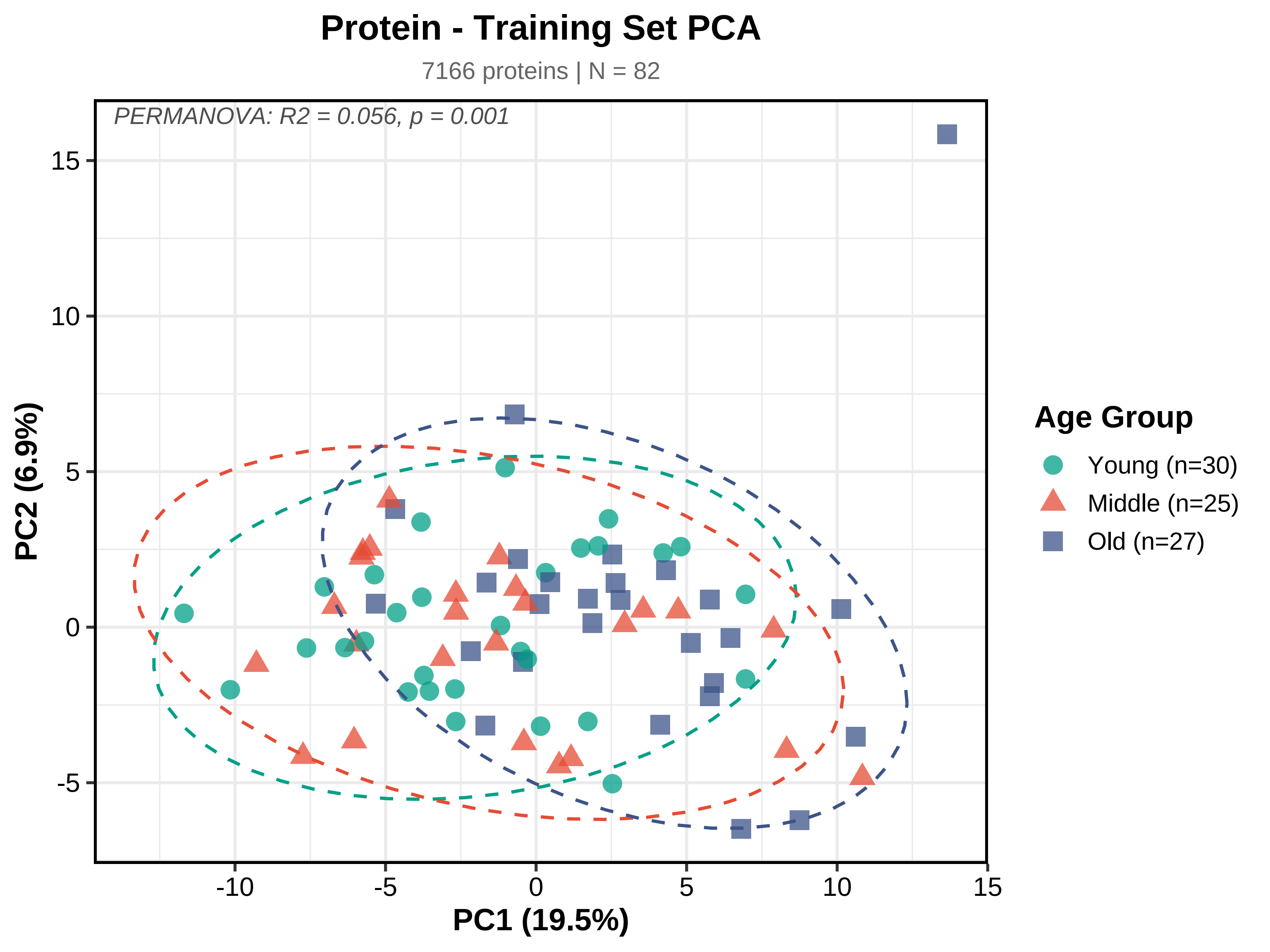
Protein - Training Set PCA
7166 proteins | N = 82
PERMANOVA: R2 = 0.056, p = 0.001
15
10
5
0
-5
-10
-5
0
5
10
15
PC2 (6.9%)
PC1 (19.5%)
Age Group
Young (n=30)
Middle (n=25)
Old (n=27)
