# Supplementary material for: Proteome-Level Autophagy–Lysosome Remodelling Marks Ageing in Human Dermal Fibroblasts and Nominates Hydroxytyrosol as a Candidate Nutraceutical
Source: Int J Mol Sci. 2026 Jul 29;27(15):6808. doi: 10.3390/ijms27156808 (PMC13467536; doi:10.3390/ijms27156808)
Supplement: Supplementary file 1 [file ijms-27-06808-s001.zip › Supplementary Figure S3a. DIABLO_plotArrow.pdf]

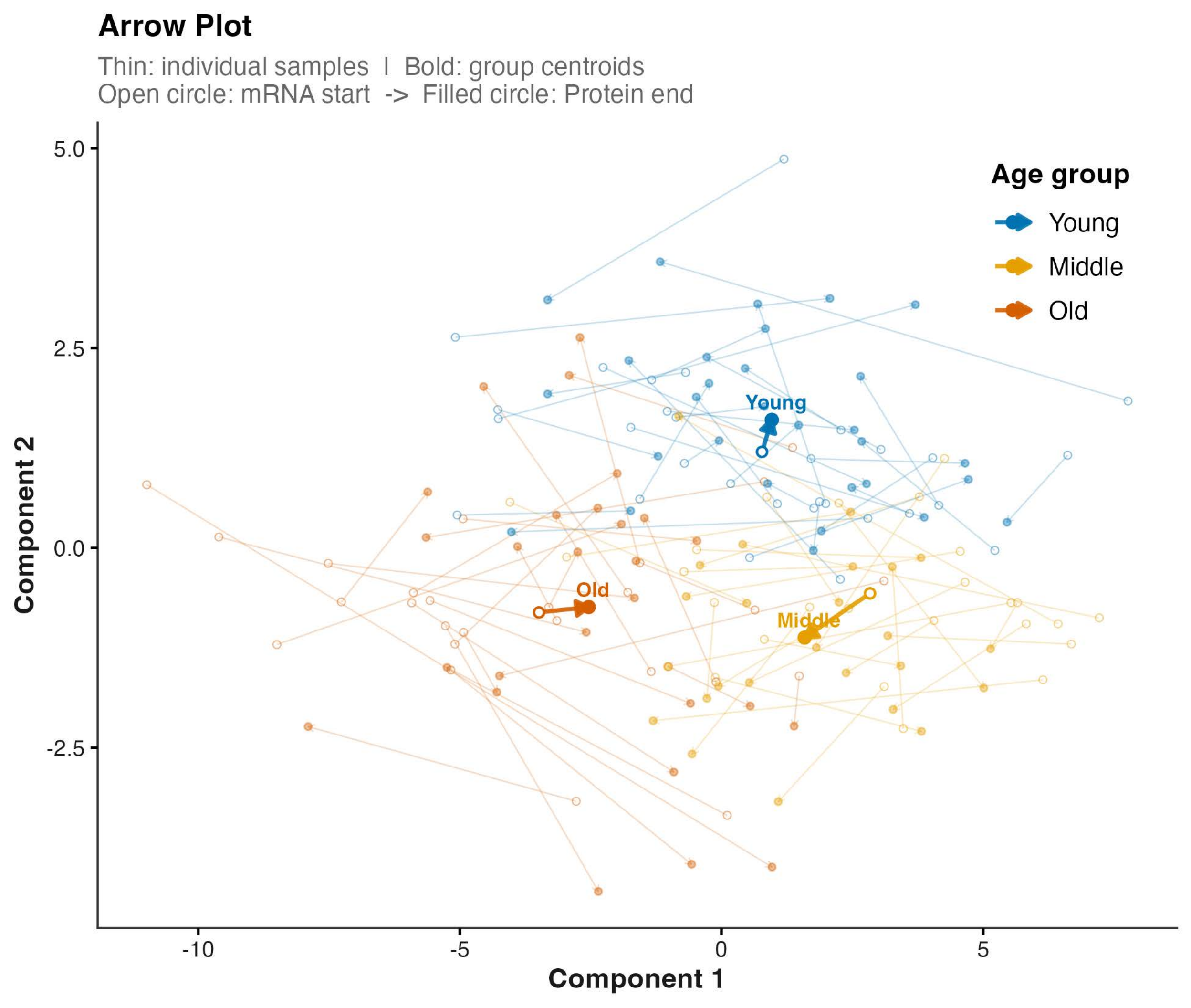

Arrow Plot
Thin: individual samples | Bold: group centroids
Open circle: mRNA start -> Filled circle: Protein end
Age group
Young
Middle
Old
Young
Middle
Old
Component 1
Component 2
5.0
2.5
0.0
-2.5
-10
-5
0
5
