# Supplementary material for: Proteome-Level Autophagy–Lysosome Remodelling Marks Ageing in Human Dermal Fibroblasts and Nominates Hydroxytyrosol as a Candidate Nutraceutical
Source: Int J Mol Sci. 2026 Jul 29;27(15):6808. doi: 10.3390/ijms27156808 (PMC13467536; doi:10.3390/ijms27156808)
Supplement: Supplementary file 1 [file ijms-27-06808-s001.zip › Supplementary Figure S3b. DIABLO_diagnostic.pdf]

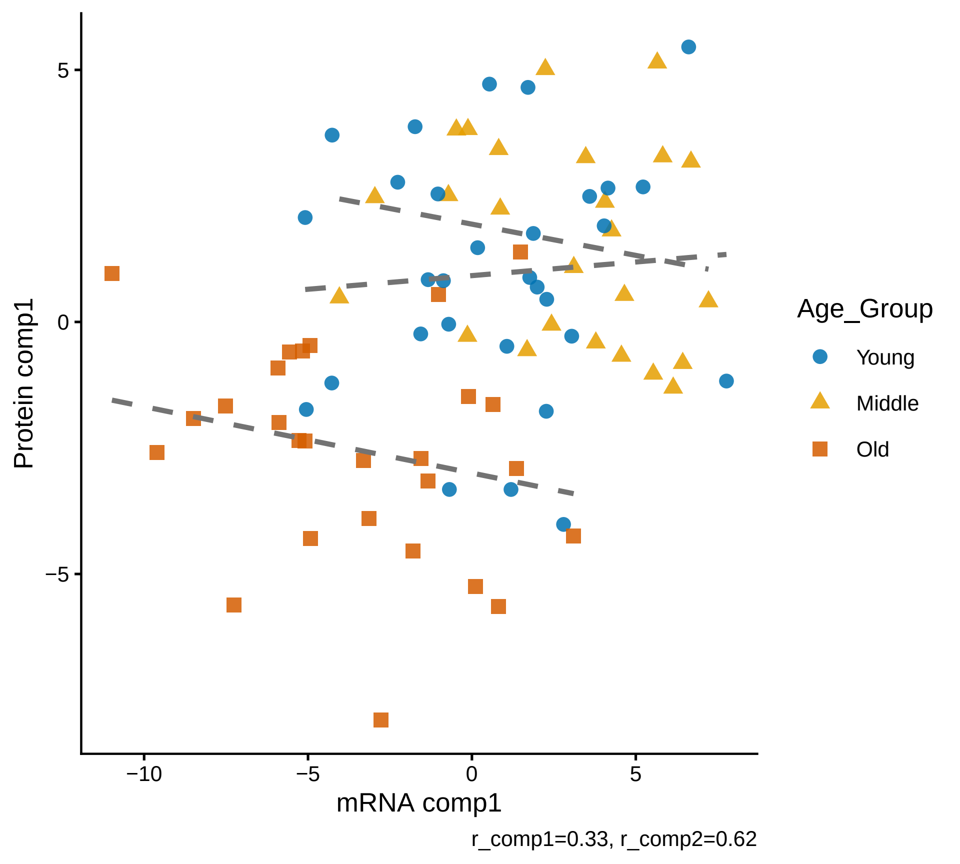

Protein comp1
5
0
−5
−10
−5
0
5
mRNA comp1
Age_Group
Young
Middle
Old
r_comp1=0.33, r_comp2=0.62
