# Supplementary material for: Proteome-Level Autophagy–Lysosome Remodelling Marks Ageing in Human Dermal Fibroblasts and Nominates Hydroxytyrosol as a Candidate Nutraceutical
Source: Int J Mol Sci. 2026 Jul 29;27(15):6808. doi: 10.3390/ijms27156808 (PMC13467536; doi:10.3390/ijms27156808)
Supplement: Supplementary file 1 [file ijms-27-06808-s001.zip › Supplementary Figure S3c. DIABLO_loadings.pdf]

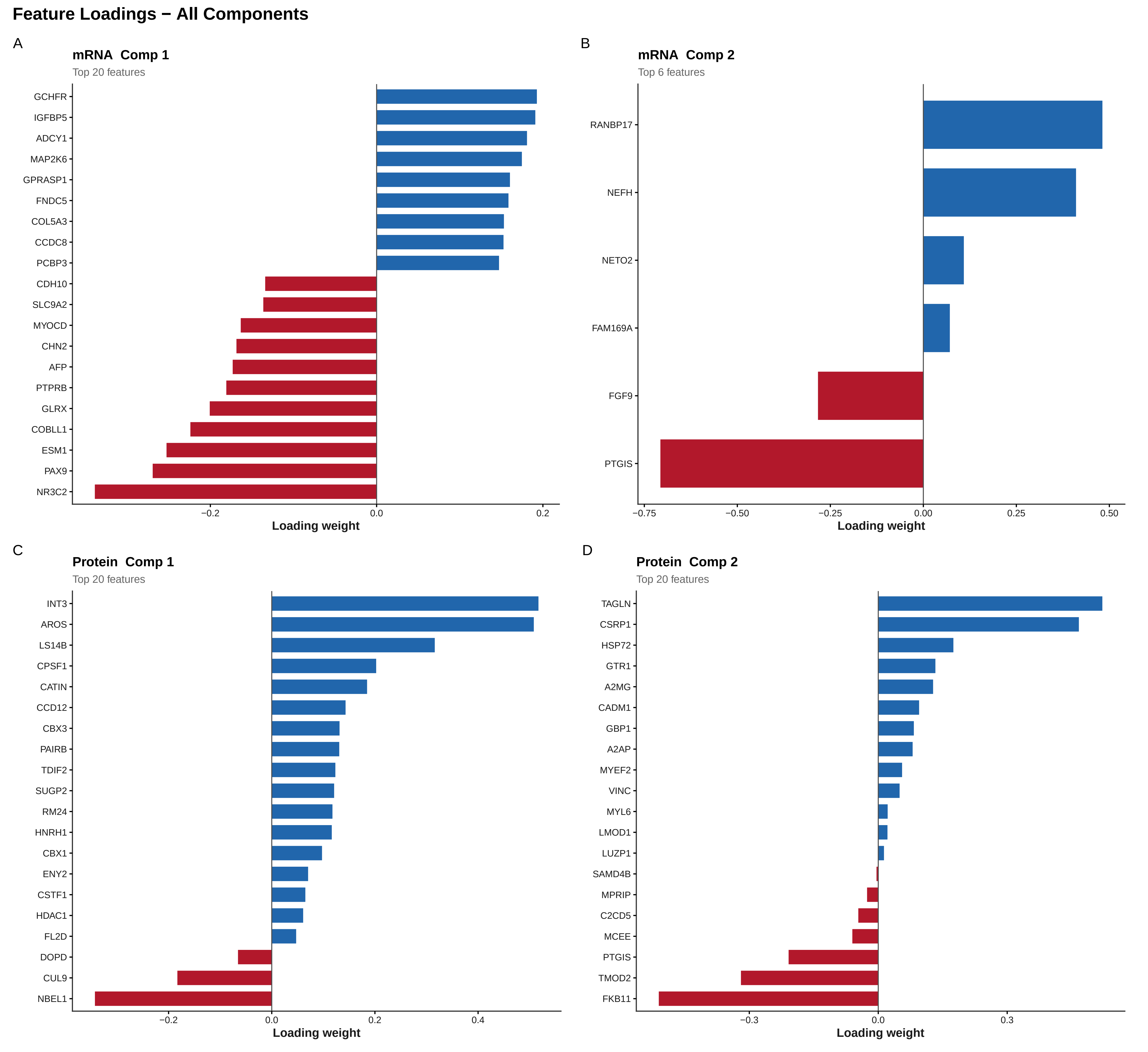
Feature Loadings − All Components
A
mRNA Comp 1
Top 20 features
GCHFR
IGFBP5
ADCY1
MAP2K6
GPRASP1
FNDC5
COL5A3
CCDC8
PCBP3
CDH10
SLC9A2
MYOCD
CHN2
AFP
PTPRB
GLRX
COBLL1
ESM1
PAX9
NR3C2
−0.2
0.0
0.2
Loading weight
B
mRNA Comp 2
Top 6 features
RANBP17
NEFH
NETO2
FAM169A
FGF9
PTGIS
−0.75
−0.50
−0.25
0.00
0.25
0.50
Loading weight
C
Protein Comp 1
Top 20 features
INT3
AROS
LS14B
CPSF1
CATIN
CCD12
CBX3
PAIRB
TDIF2
SUGP2
RM24
HNRH1
CBX1
ENY2
CSTF1
HDAC1
FL2D
DOPD
CUL9
NBEL1
−0.2
0.0
0.2
0.4
Loading weight
D
Protein Comp 2
Top 20 features
TAGLN
CSRP1
HSP72
GTR1
A2MG
CADM1
GBP1
A2AP
MYEF2
VINC
MYL6
LMOD1
LUZP1
SAMD4B
MPRIP
C2CD5
MCEE
PTGIS
TMOD2
FKB11
−0.3
0.0
0.3
Loading weight
