# Supplementary material for: Proteome-Level Autophagy–Lysosome Remodelling Marks Ageing in Human Dermal Fibroblasts and Nominates Hydroxytyrosol as a Candidate Nutraceutical
Source: Int J Mol Sci. 2026 Jul 29;27(15):6808. doi: 10.3390/ijms27156808 (PMC13467536; doi:10.3390/ijms27156808)

Feature Selection Stability

5−fold CV x 50 repeats

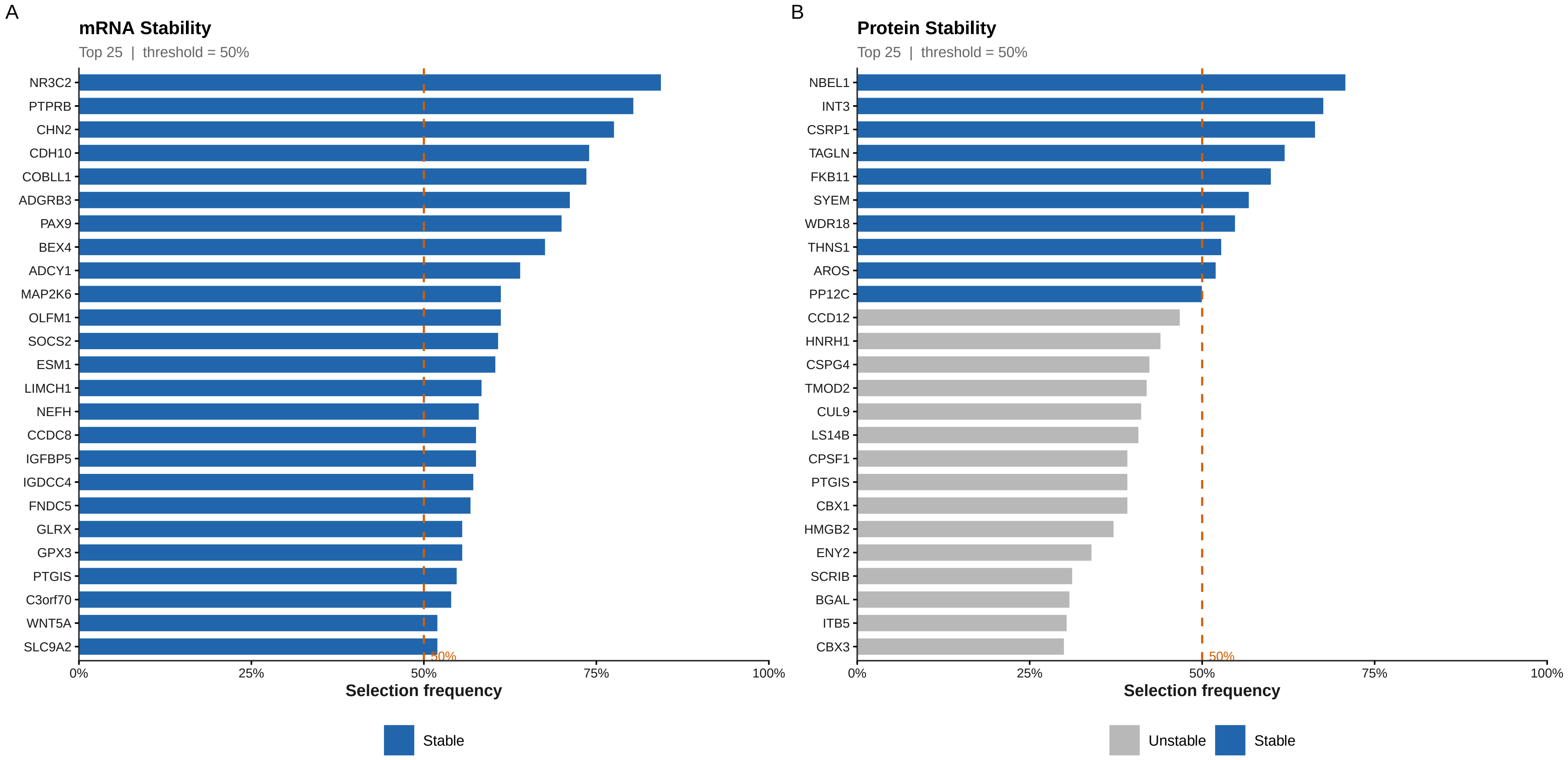

Supplement: Supplementary file 1 [file ijms-27-06808-s001.zip › Supplementary Figure S3d. DIABLO_stability.pdf]
