# Supplementary material for: Proteome-Level Autophagy–Lysosome Remodelling Marks Ageing in Human Dermal Fibroblasts and Nominates Hydroxytyrosol as a Candidate Nutraceutical
Source: Int J Mol Sci. 2026 Jul 29;27(15):6808. doi: 10.3390/ijms27156808 (PMC13467536; doi:10.3390/ijms27156808)
Supplement: Supplementary file 1 [file ijms-27-06808-s001.zip › Supplementary Figure S3e. DIABLO_permutation.pdf]

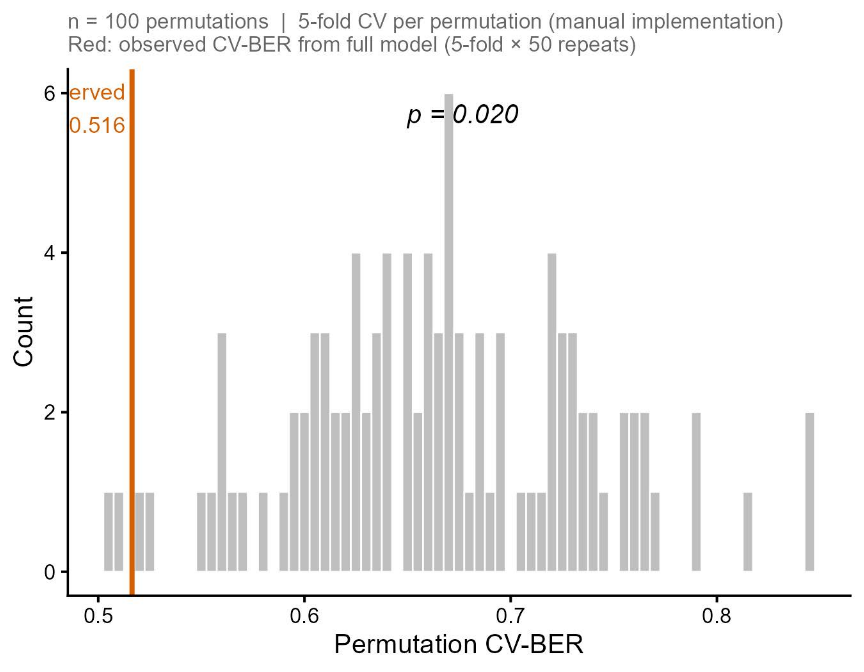
n = 100 permutations | 5-fold CV per permutation (manual implementation)
Red: observed CV-BER from full model (5-fold × 50 repeats)
erved
0.516
p = 0.020
Count
0
2
4
6
0.5
0.6
0.7
0.8
Permutation CV-BER
