# Supplementary material for: Proteome-Level Autophagy–Lysosome Remodelling Marks Ageing in Human Dermal Fibroblasts and Nominates Hydroxytyrosol as a Candidate Nutraceutical
Source: Int J Mol Sci. 2026 Jul 29;27(15):6808. doi: 10.3390/ijms27156808 (PMC13467536; doi:10.3390/ijms27156808)
Supplement: Supplementary file 1 [file ijms-27-06808-s001.zip › Supplementary Figure S4a. GSEA_GOBP_DIABLO_comp1.pdf]

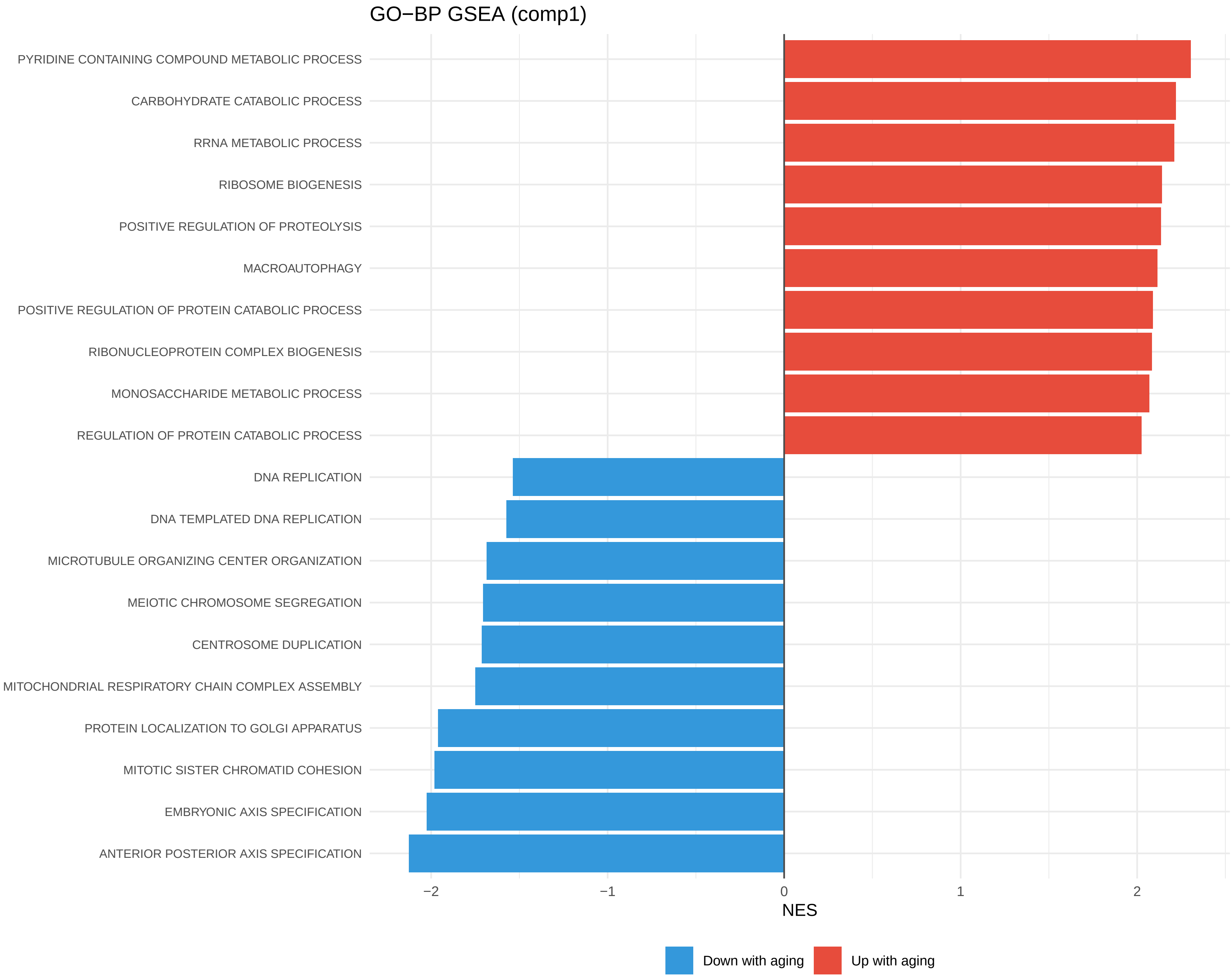
GO−BP GSEA (comp1)
PYRIDINE CONTAINING COMPOUND METABOLIC PROCESS
CARBOHYDRATE CATABOLIC PROCESS
RRNA METABOLIC PROCESS
RIBOSOME BIOGENESIS
POSITIVE REGULATION OF PROTEOLYSIS
MACROAUTOPHAGY
POSITIVE REGULATION OF PROTEIN CATABOLIC PROCESS
RIBONUCLEOPROTEIN COMPLEX BIOGENESIS
MONOSACCHARIDE METABOLIC PROCESS
REGULATION OF PROTEIN CATABOLIC PROCESS
DNA REPLICATION
DNA TEMPLATED DNA REPLICATION
MICROTUBULE ORGANIZING CENTER ORGANIZATION
MEIOTIC CHROMOSOME SEGREGATION
CENTROSOME DUPLICATION
MITOCHONDRIAL RESPIRATORY CHAIN COMPLEX ASSEMBLY
PROTEIN LOCALIZATION TO GOLGI APPARATUS
MITOTIC SISTER CHROMATID COHESION
EMBRYONIC AXIS SPECIFICATION
ANTERIOR POSTERIOR AXIS SPECIFICATION
−2
−1
0
1
2
NES
Down with aging
Up with aging
