# Supplementary material for: Proteome-Level Autophagy–Lysosome Remodelling Marks Ageing in Human Dermal Fibroblasts and Nominates Hydroxytyrosol as a Candidate Nutraceutical
Source: Int J Mol Sci. 2026 Jul 29;27(15):6808. doi: 10.3390/ijms27156808 (PMC13467536; doi:10.3390/ijms27156808)
Supplement: Supplementary file 1 [file ijms-27-06808-s001.zip › Supplementary Figure S4b. GSEA_GOBP_mRNA_age.pdf]

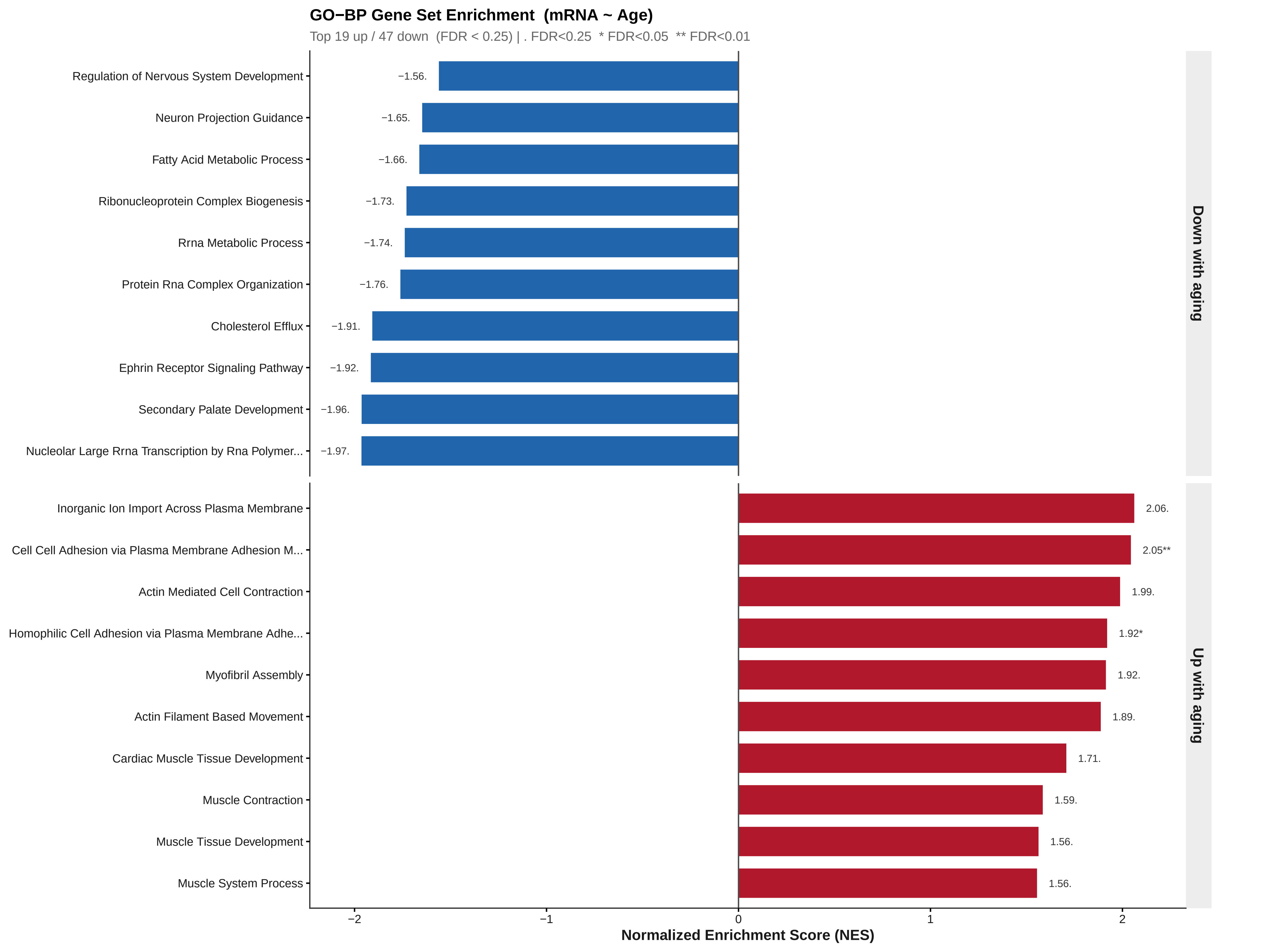
GO−BP Gene Set Enrichment (mRNA ~ Age)
Top 19 up / 47 down (FDR < 0.25) | . FDR<0.25 * FDR<0.05 ** FDR<0.01
Regulation of Nervous System Development
−1.56.
Neuron Projection Guidance
−1.65.
Fatty Acid Metabolic Process
−1.66.
Ribonucleoprotein Complex Biogenesis
−1.73.
Rrna Metabolic Process
−1.74.
Protein Rna Complex Organization
−1.76.
Cholesterol Efflux
−1.91.
Ephrin Receptor Signaling Pathway
−1.92.
Secondary Palate Development
−1.96.
Nucleolar Large Rrna Transcription by Rna Polymer...
−1.97.
Down with aging
Inorganic Ion Import Across Plasma Membrane
2.06.
Cell Cell Adhesion via Plasma Membrane Adhesion M...
2.05**
Actin Mediated Cell Contraction
1.99.
Homophilic Cell Adhesion via Plasma Membrane Adhe...
1.92*
Myofibril Assembly
1.92.
Actin Filament Based Movement
1.89.
Cardiac Muscle Tissue Development
1.71.
Muscle Contraction
1.59.
Muscle Tissue Development
1.56.
Muscle System Process
1.56.
Up with aging
−2
−1
0
1
2
Normalized Enrichment Score (NES)
