# Supplementary material for: Proteome-Level Autophagy–Lysosome Remodelling Marks Ageing in Human Dermal Fibroblasts and Nominates Hydroxytyrosol as a Candidate Nutraceutical
Source: Int J Mol Sci. 2026 Jul 29;27(15):6808. doi: 10.3390/ijms27156808 (PMC13467536; doi:10.3390/ijms27156808)
Supplement: Supplementary file 1 [file ijms-27-06808-s001.zip › Supplementary Figure S4c. GSEA_Hallmark.pdf]

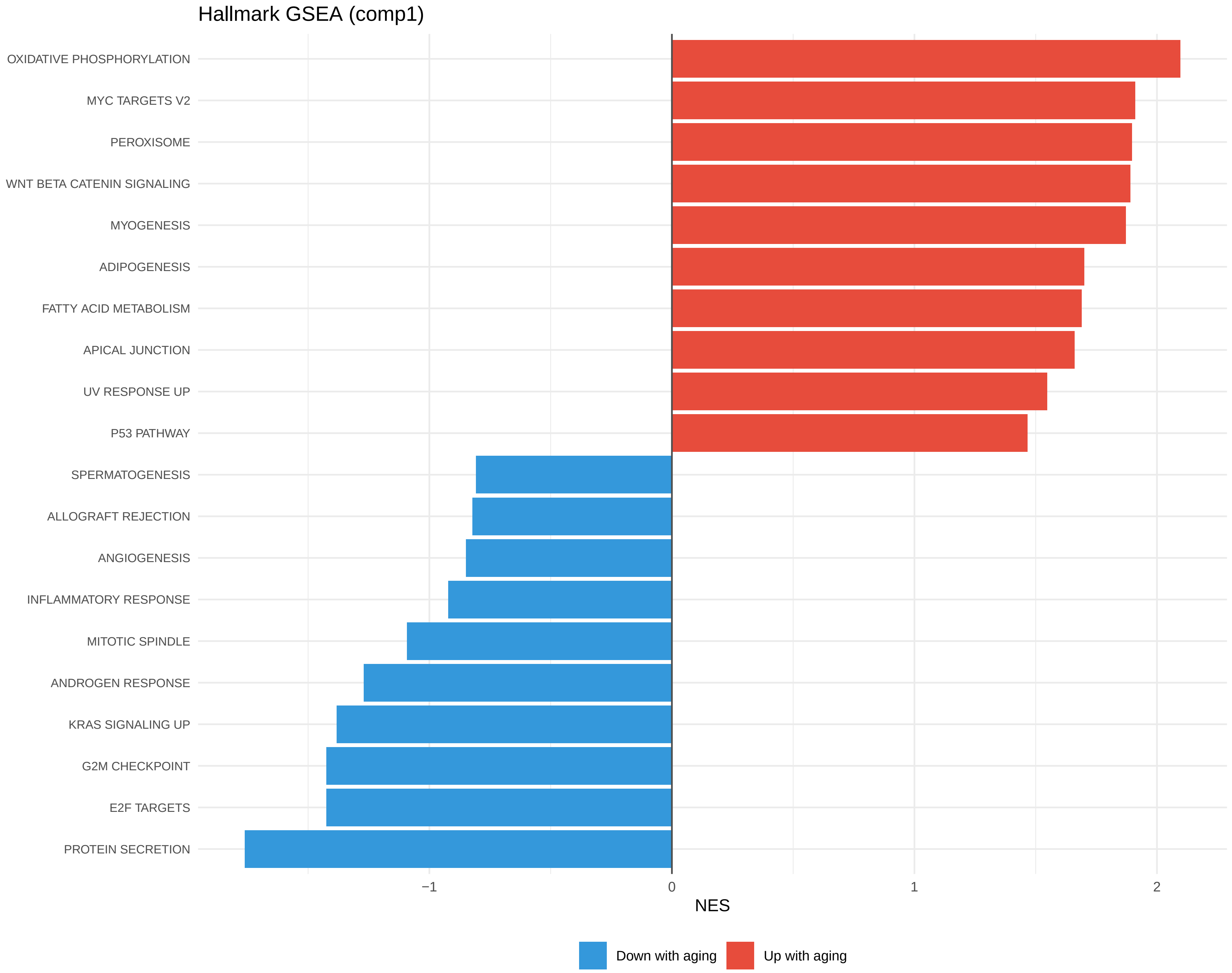
Hallmark GSEA (comp1)
OXIDATIVE PHOSPHORYLATION
MYC TARGETS V2
PEROXISOME
WNT BETA CATENIN SIGNALING
MYOGENESIS
ADIPOGENESIS
FATTY ACID METABOLISM
APICAL JUNCTION
UV RESPONSE UP
P53 PATHWAY
SPERMATOGENESIS
ALLOGRAFT REJECTION
ANGIOGENESIS
INFLAMMATORY RESPONSE
MITOTIC SPINDLE
ANDROGEN RESPONSE
KRAS SIGNALING UP
G2M CHECKPOINT
E2F TARGETS
PROTEIN SECRETION
−1
0
1
2
NES
Down with aging
Up with aging
