# Supplementary material for: Proteome-Level Autophagy–Lysosome Remodelling Marks Ageing in Human Dermal Fibroblasts and Nominates Hydroxytyrosol as a Candidate Nutraceutical
Source: Int J Mol Sci. 2026 Jul 29;27(15):6808. doi: 10.3390/ijms27156808 (PMC13467536; doi:10.3390/ijms27156808)
Supplement: Supplementary file 1 [file ijms-27-06808-s001.zip › Supplementary Figure S4d.GSEA_GOBP_Proteome.pdf]

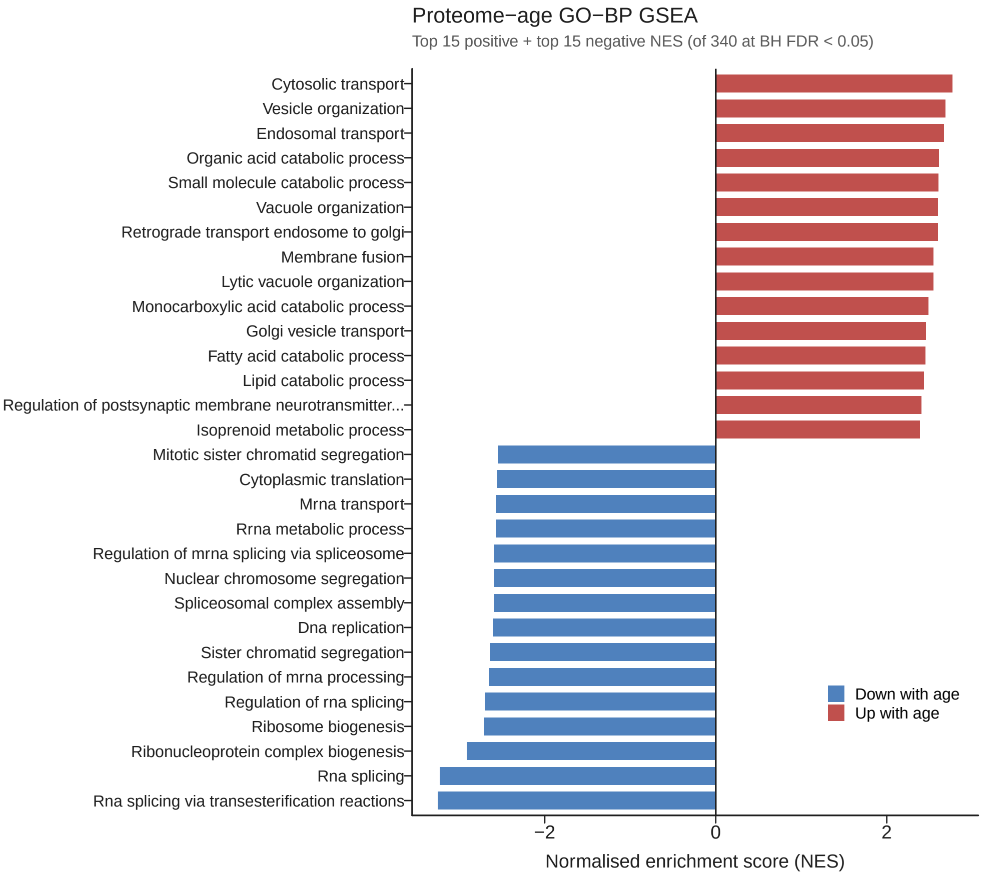
Proteome−age GO−BP GSEA
Top 15 positive + top 15 negative NES (of 340 at BH FDR < 0.05)
Cytosolic transport
Vesicle organization
Endosomal transport
Organic acid catabolic process
Small molecule catabolic process
Vacuole organization
Retrograde transport endosome to golgi
Membrane fusion
Lytic vacuole organization
Monocarboxylic acid catabolic process
Golgi vesicle transport
Fatty acid catabolic process
Lipid catabolic process
Regulation of postsynaptic membrane neurotransmitter...
Isoprenoid metabolic process
Mitotic sister chromatid segregation
Cytoplasmic translation
Mrna transport
Rrna metabolic process
Regulation of mrna splicing via spliceosome
Nuclear chromosome segregation
Spliceosomal complex assembly
Dna replication
Sister chromatid segregation
Regulation of mrna processing
Regulation of rna splicing
Ribosome biogenesis
Ribonucleoprotein complex biogenesis
Rna splicing
Rna splicing via transesterification reactions
Down with age
Up with age
−2
0
2
Normalised enrichment score (NES)
