# Supplementary material for: Proteome-Level Autophagy–Lysosome Remodelling Marks Ageing in Human Dermal Fibroblasts and Nominates Hydroxytyrosol as a Candidate Nutraceutical
Source: Int J Mol Sci. 2026 Jul 29;27(15):6808. doi: 10.3390/ijms27156808 (PMC13467536; doi:10.3390/ijms27156808)
Supplement: Supplementary file 1 [file ijms-27-06808-s001.zip › Supplementary Figure S5a. Dendrogram_modules.pdf]

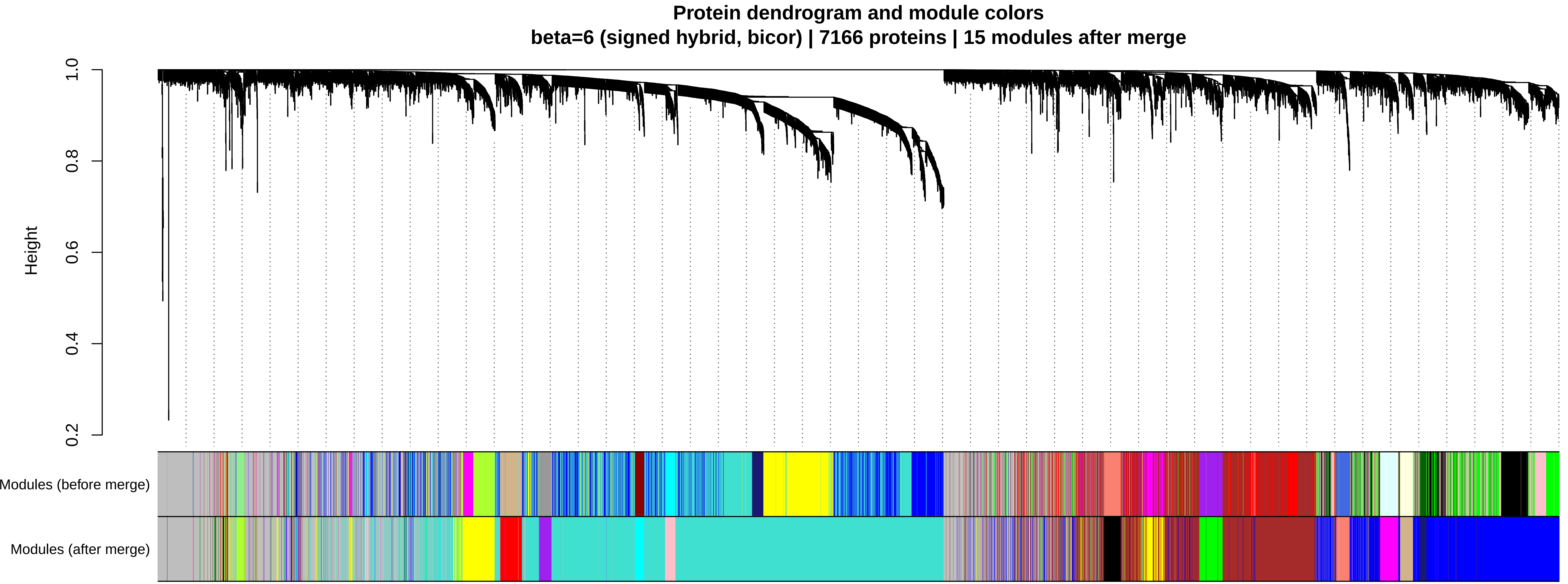
Protein dendrogram and module colors
beta=6 (signed hybrid, bicor) | 7166 proteins | 15 modules after merge
Height
1.0
0.8
0.6
0.4
0.2
Modules (before merge)
Modules (after merge)
