# Supplementary material for: Proteome-Level Autophagy–Lysosome Remodelling Marks Ageing in Human Dermal Fibroblasts and Nominates Hydroxytyrosol as a Candidate Nutraceutical
Source: Int J Mol Sci. 2026 Jul 29;27(15):6808. doi: 10.3390/ijms27156808 (PMC13467536; doi:10.3390/ijms27156808)
Supplement: Supplementary file 1 [file ijms-27-06808-s001.zip › Supplementary Figure S5b. Module_sizes.pdf]

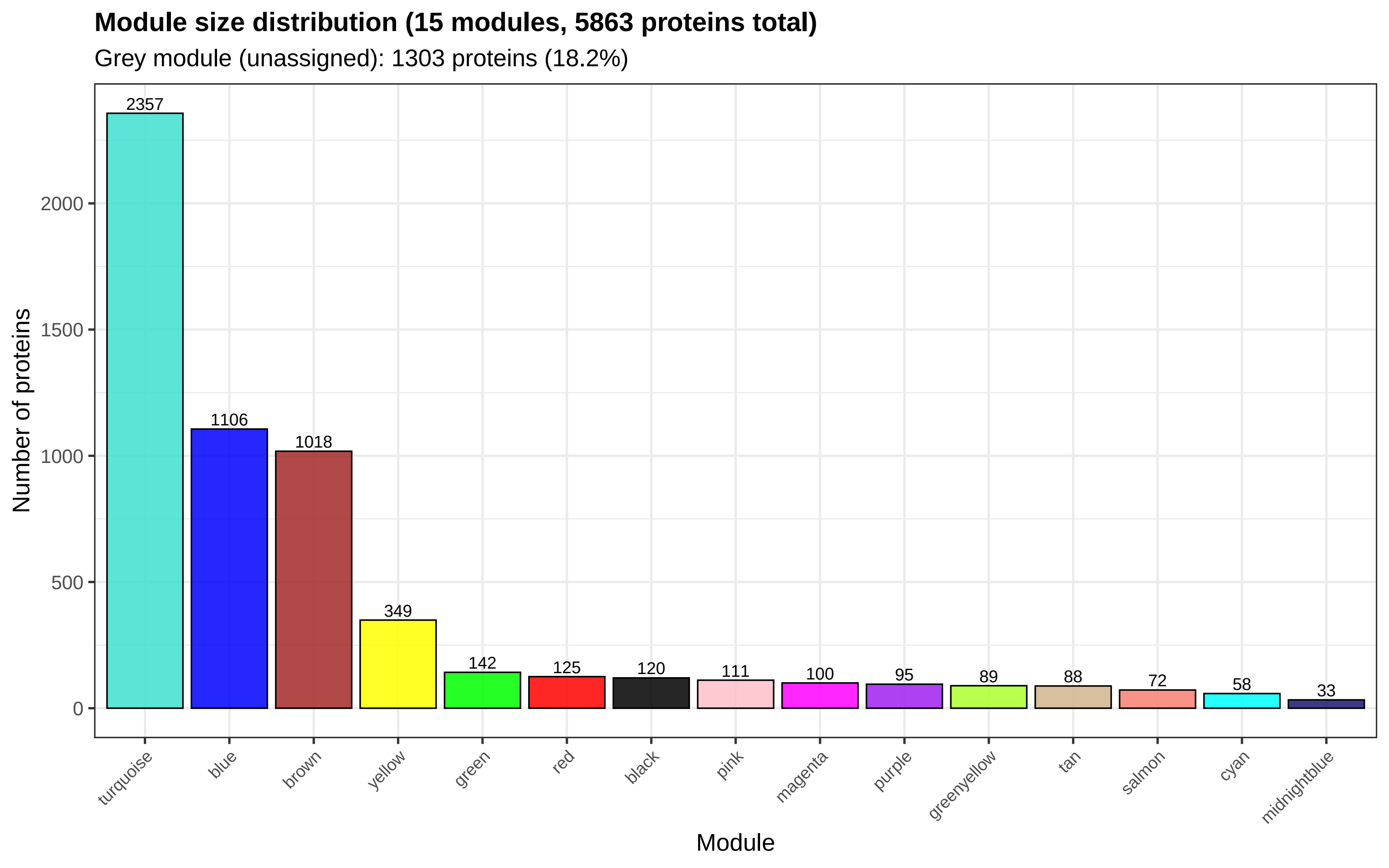
Module size distribution (15 modules, 5863 proteins total)
Grey module (unassigned): 1303 proteins (18.2%)
Number of proteins
0
500
1000
1500
2000
2357
1106
1018
349
142
125
120
111
100
95
89
88
72
58
33
turquoise
blue
brown
yellow
green
red
black
pink
magenta
purple
greenyellow
tan
salmon
cyan
midnightblue
Module
