# Supplementary material for: Proteome-Level Autophagy–Lysosome Remodelling Marks Ageing in Human Dermal Fibroblasts and Nominates Hydroxytyrosol as a Candidate Nutraceutical
Source: Int J Mol Sci. 2026 Jul 29;27(15):6808. doi: 10.3390/ijms27156808 (PMC13467536; doi:10.3390/ijms27156808)
Supplement: Supplementary file 1 [file ijms-27-06808-s001.zip › Supplementary Figure S5c. Sample_dendrogram.pdf]

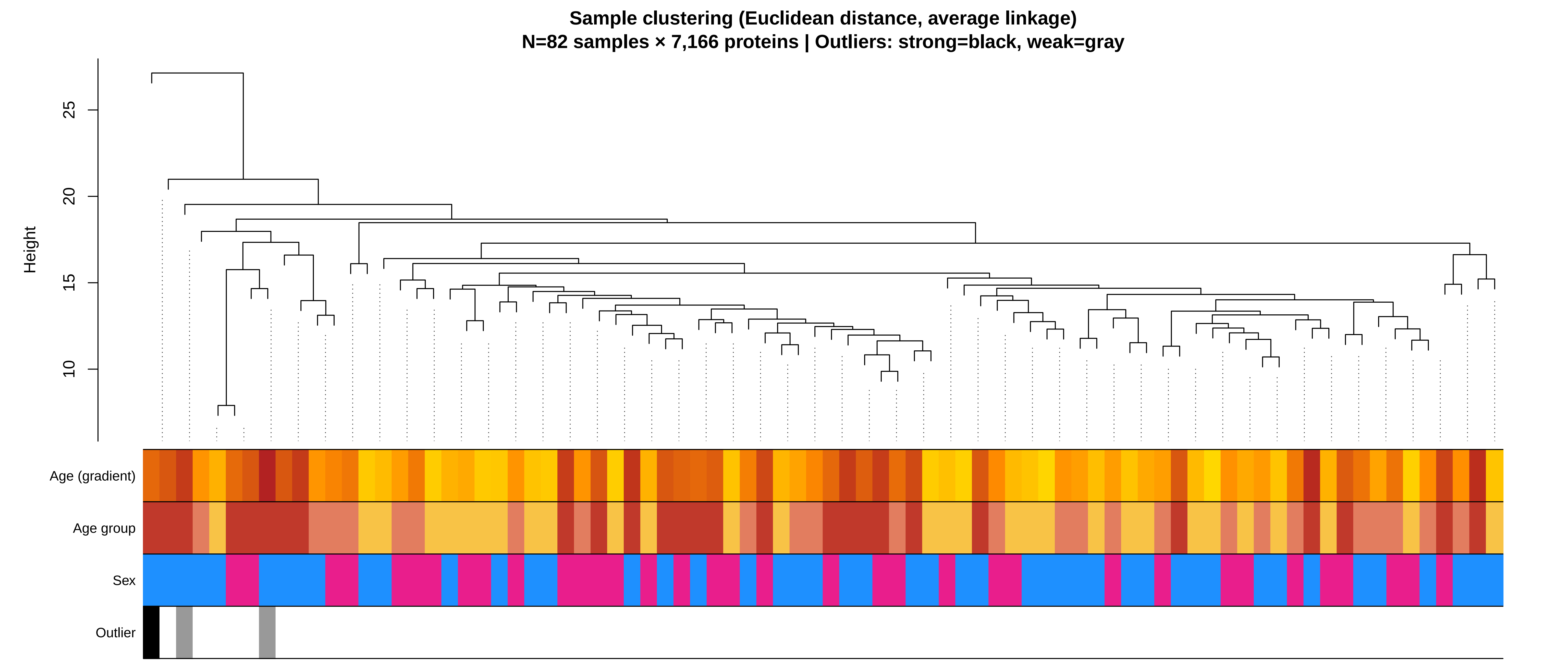

Sample clustering (Euclidean distance, average linkage)
N=82 samples × 7,166 proteins | Outliers: strong=black, weak=gray
Height
25
20
15
10
Age (gradient)
Age group
Sex
Outlier
