# Supplementary material for: Proteome-Level Autophagy–Lysosome Remodelling Marks Ageing in Human Dermal Fibroblasts and Nominates Hydroxytyrosol as a Candidate Nutraceutical
Source: Int J Mol Sci. 2026 Jul 29;27(15):6808. doi: 10.3390/ijms27156808 (PMC13467536; doi:10.3390/ijms27156808)
Supplement: Supplementary file 1 [file ijms-27-06808-s001.zip › Supplementary Figure S5d. ME_correlation.pdf]

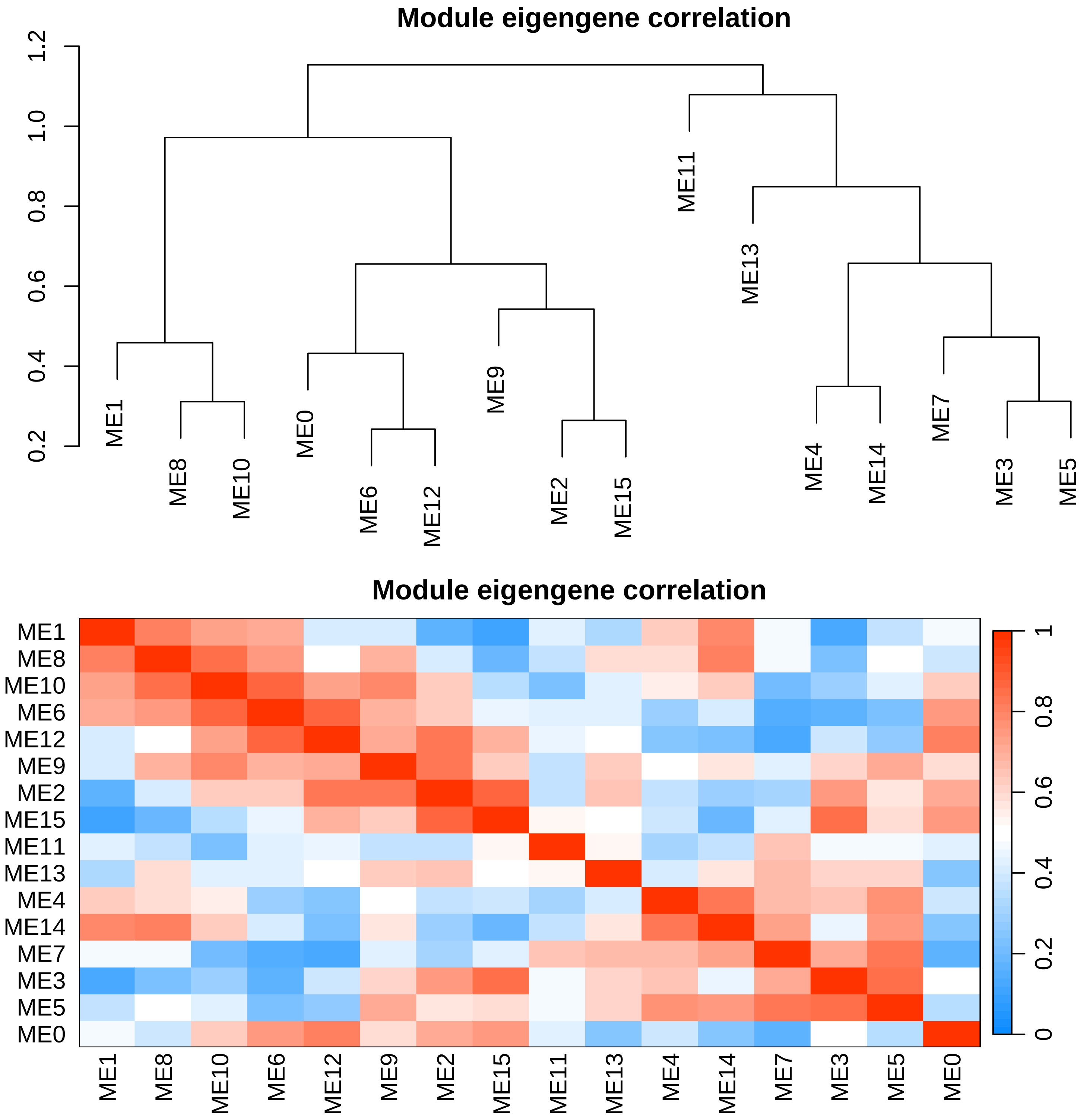

Module eigengene correlation
1.2
1.0
0.8
0.6
0.4
0.2
ME1
ME8
ME10
ME0
ME6
ME12
ME9
ME2
ME15
ME11
ME13
ME4
ME14
ME7
ME3
ME5
Module eigengene correlation
ME1
ME8
ME10
ME6
ME12
ME9
ME2
ME15
ME11
ME13
ME4
ME14
ME7
ME3
ME5
ME0
1
0.8
0.6
0.4
0.2
0
