# Supplementary material for: Proteome-Level Autophagy–Lysosome Remodelling Marks Ageing in Human Dermal Fibroblasts and Nominates Hydroxytyrosol as a Candidate Nutraceutical
Source: Int J Mol Sci. 2026 Jul 29;27(15):6808. doi: 10.3390/ijms27156808 (PMC13467536; doi:10.3390/ijms27156808)
Supplement: Supplementary file 1 [file ijms-27-06808-s001.zip › Supplementary Methods.pdf]

Supplementary Methods

Preprocessing details

For the DIABLO supervised integration, a median absolute deviation (MAD) filter was applied to the variance-stabilised transcriptomic matrix, retaining the top 8,000 most variable genes (7,976 remained after secondary biotype verification against EnsDb.Hsapiens.v86 and org.Hs.eg.db). The TMT proteomic matrix was provided by the original study after isotope impurity correction, FDR-controlled identification and global between-sample normalisation [1].

Sex correction was verified by confirming reduced feature-level sex association alongside preserved age-group F-statistics. Sample order was randomised under a fixed seed before downstream modelling. Dispersion homogeneity for the PERMANOVA was assessed with vegan::betadisper [2] (999 permutations).

Decoupling analysis: sensitivity and fallback thresholds

The DEGRADED-mode criterion was evaluated at three Pearson correlation thresholds ($r > 0.90$, 0.95 and 0.99) between the decoupling score and β_{protein}. When global transcript–protein agreement is near zero, residual-based scores collapse onto a re-ranking of β_{protein}, making the DEGRADED-mode reinterpretation necessary.

For quadrant ORA, prespecified fallback significance thresholds of FDR < 0.20 and nominal $P < 0.05$ were applied in sequence when no gene set reached the primary BH FDR < 0.05 threshold. Under DEGRADED mode, Q2 and Q4 enrichment was interpreted as protein-layer directionality rather than strict transcript–protein discordance.

Per-donor coupling index: subset definitions and additional parameters

The five prespecified gene subsets were defined as follows:

Global gene set (G): all 6,995 matched gene–protein pairs.

Autophagy execution subset (A_{exec}): Gene Toolbox autophagy core and docking/fusion categories [3].

Autophagy flux subset (A_{flux}): A_{exec} plus lysosome and mitophagy categories from the Gene Toolbox.

Broad autophagy subset (A_{broad}): full Gene Toolbox list, used as a sensitivity analysis.

Proteasomal subset (P): KEGG proteasome pathway (hsa03050) [4].

Ribosomal subset (R): KEGG ribosome pathway, used as a negative control [4].

Membership was harmonised at the HGNC symbol level; subsets with < 30 matched genes were reported descriptively only.

The decline magnitude was calculated as $\Delta\rho = \hat{\rho}_L - \hat{\rho}_H$. The sliding-window analysis excluded windows containing fewer than three donors. Bootstrap contrasts reported one-sided P values and 95% CIs for each comparison; all 12 tests were jointly BH-adjusted [5].

DIABLO tuning details

To select the design matrix, a two-block PLS was fitted on unlabelled data and the Pearson correlation of the first latent component pair (r_1) was extracted. When r_1 fell between 0.3 and 0.7, both a null design (off-diagonal = 0) and a low-covariance design (off-diagonal = 0.1) were evaluated; otherwise only the null design

was considered. Candidate designs were compared by non-sparse DIABLO under the 50-repeat cross-validation scheme; the lower-BER design was preferred, and if designs differed by less than one standard error the simpler null design was retained [6].

A second component was kept only if it reduced BER by more than 0.02. The keepX tuning grid for the transcriptome block spanned {5, 10, 15, 20, 25, 30, 35, 40, 50, 60, 70, 80} and for the proteome block {5, 10, 15, 20, 25, 30, 35, 40}; if the optimum fell on a grid boundary, the grid was expanded and tuning repeated for up to three rounds. Selection stability was classified as high-confidence (≥ 60%), medium-confidence (50–59%) or lower-confidence (< 50%).

Dual-track thresholding criteria

Track 2 candidates were selected by a tiered strategy:

Tier A: $|Z_{\text{decoupling}}| > 2$ together with proteomic age-association FDR < 0.05.

Tier B: $|Z_{\text{decoupling}}| > 2$ with proteomic FDR relaxed to 0.10.

Tier C: proteomic FDR < 0.05 with an absolute standardised age effect above an adaptive percentile threshold, lowered from the 99th toward the 50th percentile until the combined Track 1 plus Track 2 candidate set reached 80–120 features.

Under DEGRADED mode, Track 2 captured extreme protein-layer age outliers rather than strict decoupling targets. Enrichment of Track 1 proteomic features among age-associated proteins from independent linear regression was assessed by Fisher's exact test.

CES weight sensitivity

To assess the robustness of the composite evidence score (CES) ranking to the choice of weights, two alternative configurations were evaluated alongside the primary scheme (0.5, 0.3, 0.2): equal-weight (1/3, 1/3, 1/3) and stability-dominant (0.6, 0.2, 0.2). For each scheme, CES values were recomputed from the same min–max normalised inputs; under DEGRADED mode, the decoupling term remained zero in all configurations. Concordance between primary and alternative rankings was summarised by Spearman rank correlation across all dual-track candidates and by Jaccard similarity of the top-25 lists. Rankings were considered robust if all pairwise Spearman correlations exceeded 0.90.

Proteostasis module member lists

AC module. The autophagy core module followed the Gene Toolbox autophagy core category [3] and comprised the ULK complex (ULK1, ULK2, RB1CC1, ATG13, ATG101), the class III PI3K complex (PIK3C3, PIK3R4, BECN1, ATG14), WIPI1/2, ATG9A, ATG2A/B, the ATG8 conjugation machinery (ATG3, ATG5, ATG7, ATG10, ATG12, ATG16L1), the LC3/GABARAP family (MAP1LC3B, GABARAP, GABARAPL1) and the selective-autophagy receptors SQSTM1 and NBR1. When fewer than 20 of these proteins were quantified, the fusion factors STX17, SNAP29, VAMP8 and RAB7A from the Gene Toolbox docking/fusion category were added.

LC module. The lysosomal degradative-capacity module comprised 15 proteins: cathepsins CTSA, CTSB, CTSD, CTSF, CTSL and CTSS; GBA; the lysosomal membrane proteins LAMP1 and LAMP2; and V-

ATPase subunits ATP6V0A1, ATP6V0D1, ATP6V1A, ATP6V1B2, ATP6V1E1 and ATP6V1H [7]. Of the 15 members, 13 were quantified in the GESTALT proteome and used in the module-level regression.

PS module. The proteasomal module was the KEGG proteasome pathway (hsa03050) [4], including 20S core and 19S regulatory subunits. Modules with fewer than 10 quantified proteins were excluded from formal between-module testing.

McNemar test: additional specifications

The exact conditional McNemar test used two-sided *P* values from the binomial distribution of the discordant counts n_{10} (significant only in proteomics) and n_{01} (significant only in transcriptomics). When $n_{10} + n_{01} = 0$, no inferential test was performed and joint significance was reported descriptively.

WGCNA network construction and quality control

Matrix integrity was verified with goodSamplesGenes; no variance prefiltering was applied. The soft-thresholding power β was selected as the smallest value at which two consecutive R^2 increments fell below 0.005 [8 9]. Tree-cutting parameters were: maxBlockSize = 8,000, network type signed hybrid, TOM type signed [8].

Outlier detection and hub-recovery sensitivity

Outlier samples were identified by two complementary criteria: mean Euclidean distance to all other samples and Mahalanobis distance in the first five principal components. Samples flagged by both were classified as strong outliers; those flagged by only one as weak outliers. Each strong outlier triggered a paired sensitivity analysis with and without the sample. Hub recovery was defined as the fraction of primary hubs retaining hub criteria in the sensitivity network within the same-direction module (mapped by the highest ME cross-correlation). When recovery fell below 80%, hubs were reported in three tiers: robust-core (present in both networks), sensitivity-emergent and primary-only. The union of robust-core and sensitivity-emergent hubs was used for downstream interpretation.

A sensitivity grid over MM $\in \{0.80, 0.85, 0.90\}$ and $GS_{age} \in \{0.30, 0.35, 0.40\}$ was also reported. Hub tables were cross-annotated with proteomic age-regression statistics, DIABLO candidate status and membership in the AC, LC and PS modules.

Module preservation

Module preservation across age strata was evaluated by modulePreservation [10] (200 permutations) on a median-age split, with Zsummary and medianRank interpreted as within-cohort stability rather than external reproducibility.

V-ATPase pathway annotation note

Because V-ATPase subunits are annotated to several KEGG infection pathways alongside lysosomal acidification, any such enrichment hit was decomposed by overlap composition to distinguish lysosomal function from infection-pathway artefact.

GTEx preprocessing and sensitivity details

The Buja–Eyuboglu permutation procedure (sva::num.sv, method = "be", $B = 100$, vfilter = 5,000) yielded 34 surrogate variables in Suprapubic and 46 in Lower Leg [11]. After filtering and normalisation, 19,205 genes were retained in Suprapubic and 19,243 in Lower Leg. Age regression identified 1,033 age-associated transcripts in Suprapubic and 5,387 in Lower Leg (adjusted $P < 0.05$), with median $|\beta|$ of 0.001 in both tissues.

The fast-death sensitivity analysis was restricted to donors with Hardy scale 1 or 2 (Suprapubic $n = 189$, Lower Leg $n = 236$). Surrogate variables were re-estimated within each subset and the Hardy term was dropped from the design matrix. Gene-level age coefficients from the full and fast-death analyses were compared by Pearson correlation to assess robustness.

GTEx module mapping

Of the 73 GESTALT proteostasis module members (AC = 22, LC = 13, PS = 38), 72 mapped to GTEx genes retained after filtering; GBA was unrecovered in both tissues. Final GTEx module sizes were AC = 22, LC = 12 and PS = 38 in each tissue.

As a complementary test, GSVA [12] on the voom-transformed matrix derived sample-level enrichment scores for AC, LC and PS, which were correlated with midpoint-encoded age by Spearman rank correlation, with the three module-level tests BH-adjusted within each tissue.

Aging target module assembly

The Core module was the intersection of supervised DIABLO candidates and the WGCNA age-significant robust hub set. The Extended module added members supported by a single framework at prespecified secondary confidence: DIABLO selection frequency ≥ 60% not already in the Core module, or members of WGCNA age-significant modules with $GS_{age} \geq 0.30$ but module membership below the hub threshold. The 244-protein union served as the primary module; the Core module was retained as a sensitivity comparator. The LCC z-score was computed against 10,000 degree-matched random gene sets drawn from log-degree bins, with $z > 1.96$ treated as significant network agglomeration [13].

Compound library assembly and class annotation

The candidate library combined curated geroprotector entries from Geroprotectors.org [14] and DrugAge [15], supplemented by a PubMed search (1 January 2020 to 30 April 2026; Boolean query: [anti-aging OR senolytic OR senomorphic OR geroprotector OR fibroblast aging] AND [compound OR drug OR natural product]) for recent compounds with a defined PubChem CID or ChEMBL ID.

Compound class annotation used three sources: polyphenol classification from Phenol-Explorer 3.0 [16] matched by PubChem CID; food-substance status from the FDA Substances Added to Food inventory matched by normalised substance and synonym names; and a curated nutraceutical whitelist for dietary entries not covered by the first two sources.

For each compound, experimentally supported targets from ChEMBL [17] (Ki, Kd, IC50 or EC50 ≤ 10 μM), STITCH v5 [18] (combined score ≥ 0.700) and SwissTargetPrediction [19] (top-15 predicted human targets, retained without further probability cutoff per Daina et al.) were merged, deduplicated and mapped to HGNC

symbols. Compounds with fewer than 3 or more than 200 mapped targets were excluded following Guney et al. [20]. In total, 341 compounds (median 12 targets per compound) entered the proximity analysis.

Network proximity metric

The closest-distance metric of Guney et al. [20] was used:

$$d(S,T) = \frac{1}{|T|}\sum_{t\in T}\min_{s\in S} d(s,t)$$

where *S* is the aging target module and *T* the compound target set. Significance was assessed by 1,000 random reference sets matched in size and degree distribution, sampled from log-degree bins [13]. The observed distance was converted to a *z*-score; $z \le -0.15$ was recorded as proximal and $z \le -2$ as stringent proximity.

Direct overlap with the primary module was tested by one-sided Fisher's exact test against the STRING-LCC background (N = 15,882), BH-corrected across compounds [5].

CPS weight sensitivity

Following the composite reranking framework of Karatzas et al. [21], robustness of the CPS ranking was probed across three alternative weighting schemes: proximity-heavy (0.6, 0.3, 0.1), ORA–proximity-equal (0.4, 0.4, 0.2) and uniform (1/3, 1/3, 1/3). Concordance was summarised by Spearman correlation of CPS ranks and Jaccard similarity of top-*k* lists.

Nutraceutical enrichment and pathway analysis

Enrichment of nutraceuticals among top-*k* cohorts (k = 10, 25, 50, 100) was tested by one-sided Fisher's exact test against the 17.0% nutraceutical background. Pathway ORA on the union target set of Level I + II compounds against the STRING-LCC background used fgsea::fora (v1.30.0) [22] with GO Biological Process and KEGG sets from MSigDB v7.5.1 (background sizes 5–1,000; GO-BP n = 7,272; KEGG n = 186). Autophagy, lysosome, mTOR signalling, cellular senescence, insulin/IGF, ER UPR and the ubiquitin–proteasome system were prespecified as hallmark-of-aging-relevant pathway families [23].

Sub-module member lists and niche definitions

$\mathbf{M_{LC}}$ comprised 15 proteins: cathepsins CTSA, CTSB, CTSD, CTSF, CTSL and CTSS; GBA; LAMP1 and LAMP2; V-ATPase subunits ATP6V0A1, ATP6V0D1, ATP6V1A, ATP6V1B2, ATP6V1E1 and ATP6V1H [7].

$\mathbf{M_{AC}}$ comprised 29 proteins from the Gene Toolbox autophagy core and docking/fusion categories [3]: the ULK complex (ULK1, ULK2, RB1CC1, ATG13, ATG101), the class III PI3K complex (PIK3C3, PIK3R4, BECN1, ATG14), WIPI1/2, ATG9A, ATG2A/B, the ATG8 conjugation machinery (ATG3, ATG5, ATG7, ATG10, ATG12, ATG16L1), the LC3/GABARAP family (MAP1LC3B, GABARAP, GABARAPL1), SQSTM1/NBR1, and STX17, SNAP29, VAMP8 and RAB7A.

$\mathbf{M_{chromatin}}$ comprised 21 proteins: the four Core-module hubs (CBX3, CBX5, HNRNPH1, SUGP2) and their first-shell interactome partners HDAC1/2, DNMT1, SMARCA4, RBBP7, RCOR1, CHAF1A/B, CHAMP1, ADNP, EWSR1, FUS, TIA1, NELFE, SRSF7, CSTF2 and CBX1. Each sub-module was projected onto the STRING-LCC; mapping rates exceeded 95% for all three.

Niche assignment rules. LC-preferential: $z_{\mathrm{LC}} \leq -2$, $z_{\mathrm{AC}} > -2$ and $z_{\mathrm{chromatin}} > -2$. AC-preferential and chromatin-preferential: defined analogously. Autophagy–lysosome-broad: both $z_{\mathrm{LC}} \leq -2$ and $z_{\mathrm{AC}} \leq -2$ regardless of $z_{\mathrm{chromatin}}$. Broad-coverage: all three $z \leq -2$. Non-specific: none reached $z \leq -2$. Compounds with $|T| < 10$ were flagged as low-power candidates.

Pairwise separation and topology classification

Pairwise network-based separation s_{AB} was computed among all top-25 nutraceutical pairs [13, 24]:

$$s_{AB} = \langle d_{AB} \rangle - \frac{\langle d_{AA} \rangle + \langle d_{BB} \rangle}{2}$$

where $\langle d_{\mathrm{AA}} \rangle$ and $\langle d_{\mathrm{BB}} \rangle$ are mean closest-path distances within each compound target set and $\langle d_{\mathrm{AB}} \rangle$ is the mean between sets. Cheng et al. partitioned drug–drug–disease topologies into six classes (P1 Overlapping Exposure through P6 Independent Action) [24]; only the P2 Complementary Exposure class was statistically associated with combination efficacy. Each pair was classified using single-compound z-scores and s_{AB}, with per-pair degree-corrected z-scores estimated against 200 random pairs. Class distribution, Fisher's exact test for over-representation of any single compound as a P2 anchor, and correspondence between P2 pairs and sub-module assignments were reported.

Molecular docking: additional preparation details

Protein structures were prepared by removing water molecules, co-crystallised ligands and non-standard residues, followed by addition of polar hydrogen atoms and CHARMm force-field typing [25]. PubChem CIDs for the six compounds: Hydroxytyrosol (82755), Kaempferol (5280863), Resveratrol (445154), Luteolin (5280445), Tyrosol (10393) and Isorhamnetin (5281654). Ligand conformations were retrieved from PubChem, prepared using the Prepare Ligands protocol and energy-minimised with the CHARMm force field.

Extended Data Figure Legends

Extended Data Fig. 1 | GO Biological Process over-representation analysis of the two discordant decoupling quadrants. GO Biological Process terms enriched among (a) Q2 genes, whose transcripts fall while their proteins rise with age (protein-accumulating), and (b) Q4 genes, whose transcripts rise while their proteins fall with age (protein-depleting). Enrichment was tested with fgsea::fora against the matched gene-protein background. Each panel shows the 15 most significant terms remaining after Wang semantic-similarity redundancy reduction (threshold 0.70). Point position gives fold enrichment, point area the number of overlapping genes, and fill the −log10 BH-adjusted P value; the dashed line marks a fold enrichment of 1. All terms shown satisfy BH FDR < 0.05.

Extended Data Fig. 2 | Principal component analysis of the transcriptomic and proteomic matrices by age group. Donor scores on the first two principal components of (a) the sex-corrected transcriptome (7,976 genes) and (b) the sex-corrected proteome (7,166 proteins), for the 82 GESTALT dermal fibroblast donors. Points are coloured by age tertile (Young, 22–42 years, n = 30; Middle, 43–65 years, n = 25; Old, 66–89 years, n = 27), and dashed ellipses outline each group. Inset text gives the PERMANOVA effect size and P value for age-group separation (Euclidean distance, 999 permutations).

Extended Data Fig. 3 | DIABLO supervised multi-omics integration. (a) Arrow plot of the fitted DIABLO space: each thin segment joins a donor's transcriptomic position (open circle) to its proteomic position (filled circle), and bold segments mark the age-group centroids, coloured by age group. (b) Donor scores on transcriptomic component 1 against proteomic component 1, coloured by age group; inset text gives the between-block Pearson correlation for components 1 and 2. (c) Variables carrying the largest absolute loadings on components 1 and 2 in the transcriptomic and proteomic blocks, with bar direction showing the sign of the loading. (d) Cross-validation selection frequency of the top 25 transcriptomic and proteomic features (5-fold cross-validation, 50 repeats); the dashed line marks the 50% stability threshold.

Extended Data Fig. 4 | Gene-set enrichment analysis across component- and age-based feature rankings. Bars give normalised enrichment scores (NES), coloured by the direction of change with age. (a) GO Biological Process gene sets tested against transcriptomic features ranked by their association with DIABLO component 1. (b) GO Biological Process gene sets tested against transcriptomic features ranked by univariate age association; symbols denote BH FDR (. < 0.25, * < 0.05, ** < 0.01), and the panel shows the 19 positive and 47 negative sets at FDR < 0.25. (c) MSigDB Hallmark gene sets tested against the same DIABLO component 1 ranking as in (a). (d) GO Biological Process gene sets tested against proteomic features ranked by age association, showing the 15 highest and 15 lowest NES among 340 sets at BH FDR < 0.05.

Extended Data Fig. 5 | Weighted gene co-expression network analysis of the GESTALT proteome. (a) Hierarchical clustering of the 7,166 proteins on topological overlap dissimilarity (soft-thresholding power β = 6, signed hybrid network, biweight midcorrelation); colour bars below the dendrogram show module assignment before and after module merging, leaving 15 modules. (b) Number of proteins per module; a further 1,303 proteins (18.2%) remained unassigned (grey). (c) Hierarchical clustering of the 82 samples on

Euclidean distance, annotated with age, age group, sex and outlier status, used to screen outliers before network construction. (d) Dendrogram and cross-correlation heatmap of the module eigengenes.

References

1 Tsitsipatis, D. *et al.* Proteomes of primary skin fibroblasts from healthy individuals reveal altered cell responses across the life span. *Aging Cell* **21**, e13609 (2022). https://doi.org/10.1111/acel.13609

2 Anderson, M. J. Distance-based tests for homogeneity of multivariate dispersions. *Biometrics* **62**, 245-253 (2006). https://doi.org/10.1111/j.1541-0420.2005.00440.x

3 Bordi, M. *et al.* A gene toolbox for monitoring autophagy transcription. *Cell Death Dis* **12**, 1044 (2021). https://doi.org/10.1038/s41419-021-04121-9

4 Kanehisa, M., Furumichi, M., Sato, Y., Kawashima, M. & Ishiguro-Watanabe, M. KEGG for taxonomy-based analysis of pathways and genomes. *Nucleic Acids Res* **51**, D587-D592 (2023). https://doi.org/10.1093/nar/gkac963

5 Benjamini, Y. & Hochberg, Y. Controlling the False Discovery Rate: A Practical and Powerful Approach to Multiple Testing. *Journal of the Royal Statistical Society Series B: Statistical Methodology* **57**, 289-300 (1995). https://doi.org/10.1111/j.2517-6161.1995.tb02031.x

6 Singh, A. *et al.* DIABLO: an integrative approach for identifying key molecular drivers from multi-omics assays. *Bioinformatics* **35**, 3055-3062 (2019). https://doi.org/10.1093/bioinformatics/bty1054

7 Forgac, M. Vacuolar ATPases: rotary proton pumps in physiology and pathophysiology. *Nat Rev Mol Cell Biol* **8**, 917-929 (2007). https://doi.org/10.1038/nrm2272

8 Zhang, B. & Horvath, S. A general framework for weighted gene co-expression network analysis. *Stat Appl Genet Mol Biol* **4**, Article17 (2005). https://doi.org/10.2202/1544-6115.1128

9 Langfelder, P. & Horvath, S. WGCNA: an R package for weighted correlation network analysis. *BMC Bioinformatics* **9**, 559 (2008). https://doi.org/10.1186/1471-2105-9-559

10 Langfelder, P., Luo, R., Oldham, M. C. & Horvath, S. Is my network module preserved and reproducible? *PLoS Comput Biol* **7**, e1001057 (2011). https://doi.org/10.1371/journal.pcbi.1001057

11 Leek, J. T., Johnson, W. E., Parker, H. S., Jaffe, A. E. & Storey, J. D. The sva package for removing batch effects and other unwanted variation in high-throughput experiments. *Bioinformatics* **28**, 882-883 (2012). https://doi.org/10.1093/bioinformatics/bts034

12 Hanzelmann, S., Castelo, R. & Guinney, J. GSVA: gene set variation analysis for microarray and RNA-seq data. *BMC Bioinformatics* **14**, 7 (2013). https://doi.org/10.1186/1471-2105-14-7

13 Menche, J. *et al.* Disease networks. Uncovering disease-disease relationships through the incomplete interactome. *Science* **347**, 1257601 (2015). https://doi.org/10.1126/science.1257601

14 Moskalev, A. *et al.* Geroprotectors.org: a new, structured and curated database of current therapeutic interventions in aging and age-related disease. *Aging (Albany NY)* **7**, 616-628 (2015). https://doi.org/10.18632/aging.100799

15 Barardo, D., Thornton, D., Thoppil, H. & et al. The DrugAge database of aging-related drugs. *Aging Cell* **16**, 594-597 (2017). https://doi.org/10.1111/acel.12585

16 Rothwell, J. A. *et al.* Phenol-Explorer 3.0: a major update of the Phenol-Explorer database to incorporate data on the effects of food processing on polyphenol content. *Database (Oxford)* **2013**, bat070 (2013). https://doi.org/10.1093/database/bat070

17 Mendez, D. *et al.* ChEMBL: towards direct deposition of bioassay data. *Nucleic Acids Res* **47**, D930-D940 (2019). https://doi.org/10.1093/nar/gky1075

18 Szklarczyk, D. *et al.* STITCH 5: augmenting protein-chemical interaction networks with tissue and affinity data. *Nucleic Acids Res* **44**, D380-384 (2016). https://doi.org/10.1093/nar/gkv1277

19 Daina, A., Michielin, O. & Zoete, V. SwissTargetPrediction: updated data and new features for efficient prediction of protein targets of small molecules. *Nucleic Acids Res* **47**, W357-W364 (2019). https://doi.org/10.1093/nar/gkz382

20 Guney, E., Menche, J., Vidal, M. & Barabasi, A. L. Network-based in silico drug efficacy screening. *Nat Commun* **7**, 10331 (2016). https://doi.org/10.1038/ncomms10331

21 Karatzas, E., Kolios, G. & Spyrou, G. M. A web tool for ranking candidate drugs against a selected disease based on a combination of functional and structural criteria. *Computational and Structural Biotechnology Journal* **17**, 939-945 (2019). https://doi.org/10.1016/j.csbj.2019.05.004

22 Korotkevich, G. et al. Fast gene set enrichment analysis. Preprint at bioRxiv https://doi.org/10.1101/060012 (2021).

23 López-Otín, C., Blasco, M. A., Partridge, L., Serrano, M. & Kroemer, G. Hallmarks of aging: An expanding universe. *Cell* **186**, 243-278 (2023). https://doi.org/10.1016/j.cell.2022.11.001
24 Cheng, F., Kovacs, I. A. & Barabasi, A. L. Network-based prediction of drug combinations. *Nat Commun* **10**, 1197 (2019). https://doi.org/10.1038/s41467-019-09186-x
25 Brooks, B. R. *et al.* CHARMM: The biomolecular simulation program. *Journal of Computational Chemistry* **30**, 1545-1614 (2009). https://doi.org/10.1002/jcc.21287
